# Prophylactic Interventions for Hereditary Breast and Ovarian Cancer Risks and Mortality in BRCA1/2 Carriers

**DOI:** 10.3390/cancers16010103

**Published:** 2023-12-24

**Authors:** Taoran Liu, Jing Yu, Yangyang Gao, Xinyang Ma, Shan Jiang, Yuanyuan Gu, Wai-kit Ming

**Affiliations:** 1 Department of Infectious Diseases and Public Health, City University of Hong Kong, Hong Kong 999077, China; 2 Macquarie University Centre for the Health Economy, Macquarie Business School and Australian Institute of Health Innovation, Macquarie University, Sydney, NSW 2109, Australia

**Keywords:** hereditary breast and ovarian cancer, cancer prevention, precision medicine, systematic review, meta-analysis, chemoprevention, BRCA, genomics, genetics, precision public health

## Abstract

**Simple Summary:**

Prophylactic interventions are critical for BRCA1/2 mutation carriers to reduce the risks of hereditary breast and ovarian cancer (HBOC). This systematic review and meta-analysis synthesizes data on the efficacy of various preventative strategies. The analysis incorporated 21 studies involving risk-reducing surgeries (RRS), chemoprevention, and screening. Findings indicate a substantial reduction in breast and ovarian cancer incidence and mortality among BRCA1/2 carriers following RRS, particularly mastectomy and oophorectomy, compared to chemoprevention. This study underscores the necessity of tailored interventions, especially considering the varied protective effects between BRCA1 and BRCA2 mutation carriers. This review aids in informing clinical decisions and underscores the importance of context-specific uptake rates in cost-effective evaluations.

**Abstract:**

Background: Hereditary breast and ovarian cancers (HBOCs) pose significant health risks worldwide and are mitigated by prophylactic interventions. However, a meta-analysis of their efficacy and the impact of different genetic variants on their effectiveness is lacking. Methods: A systematic review and meta-analysis were conducted, adhering to Cochrane guidelines. The review encompassed studies that involved prophylactic interventions for healthy women with BRCA variants, focusing on cancer incidence and mortality outcomes. The Newcastle–Ottawa Scale was used for risk of bias assessment. We pooled the extracted outcomes using random effects models and conducted subgroup analyses stratified by intervention, variant, and cancer types. Results: A total of 21 studies met the inclusion criteria. The meta-analysis revealed that prophylactic interventions significantly reduced cancer risk and mortality. The subgroup analysis showed a greater protective effect for BRCA2 than BRCA1 variant carriers. Risk-reducing surgeries (RRS) were more effective than chemoprevention, with RRS notably reducing cancer risk by 56% compared to 39% for chemoprevention. Prophylactic oophorectomy significantly reduced HBOC risks, while the effect of prophylactic mastectomy and chemoprevention on mortality was less conclusive. Conclusions: Prophylactic interventions significantly reduce the risk of HBOC and associated mortality. This comprehensive analysis provides insights for future economic evaluations and clinical decision-making in HBOC interventions.

## 1. Introduction

Breast and ovarian cancers are among the leading causes of mortality worldwide. In 2020, around 2.3 million women were diagnosed with breast cancer [1] and 0.3 million women were diagnosed with ovarian cancer [2]. The average lifetime risks for women of developing breast cancer and ovarian cancer are 1 in 8 and 1 in 78, respectively [1,3]; however, studies have shown that women with inherited breast cancer 1 gene (BRCA1) and breast cancer 2 gene (BRCA2) carriers have substantially increasing lifetime risk of developing breast cancer and ovarian cancer, with a lifetime risk of 56% to 84% for breast cancer [4,5,6,7] and 36% to 63% for ovarian cancer for BRCA1 variant carriers and 10% to 27% for BRCA2 carriers [4,5,8,9,10].

There are various risk management strategies for healthy BRCA1/2 variant carriers, including risk-reducing mastectomy (RRM), risk-reducing salpingo-oophorectomy (RRSO), chemoprevention (e.g., tamoxifen), and screening (annual MRI, annual mammography, self-breast examination, etc.). Screening options and genetic testing are served to maximize the early detection of elevated risks of gynecologic cancers for BRCA1/2 carriers and thus provide insights for choosing risk-reducing strategies. Also, genetic testing significantly motivates BRCA1/2 carriers to take further prophylactic interventions, which has been summarized in a study by Metcalfe et al. [11], who found that the rate of taking RRM and breast MRI was significantly improved in carriers who received genetic testing more recently than those who tested more than 10 years ago. Although genetic testing and screening elevated the uptake rate of further interventions, they cannot prevent the development of disease. For ovarian cancer prevention, RRSO stands as the primary surgical strategy, markedly decreasing overall mortality by 60% and ovarian cancer-specific mortality by 79% [10,11,12]. Among BRCA variant carriers, the uptake for RRSO has been documented to be as high as 64.7% [11]. In addressing breast cancer risk, RRM is associated with an exceptional reduction rate exceeding 90%, positioning it as the most effective prophylactic measure for breast cancer risk reduction. BRCA carriers reached 28% and are advised and undergo RRM. As an alternative prophylactic option for risk reduction in breast cancer, chemoprevention has been relatively less mentioned. Around 6.3% of BRCA carriers decide to undergo chemoprevention, such as tamoxifen or raloxifene. These selective estrogen receptor modulators (SERMs), especially tamoxifen offer, a breast cancer risk reduction of about 48% for high-risk individuals, while raloxifene achieves a 25% incidence reduction [12].

These prophylactic risk-reduction interventions, mentioned in prior research, have demonstrated a substantial reduction in the incidence of cancers. However, there has been a lack of systematic reviews and comparisons regarding the variability of these prophylactic options, especially among different types of risk-reducing surgeries (RRS) or between RRS and chemoprevention. Moreover, advancements in screening modalities, especially with MRI, have not been comprehensively explored in the existing literature. Therefore, updated information is required to improve the basis of informed counseling for prophylactic surgeries and chemoprevention, especially from the dimension of updated uptake rates and efficacy of RRS, chemoprevention, and screening programs. Hence, this study aims to provide a systematic review encompassing RRSO, RRM, and the latest in screening and chemoprevention and offer updated information in aiding BRCA1/2 carriers’ and their clinicians’ decision-making when choosing further prophylactic options.

## 2. Methods

### 2.1. Search Strategy and Inclusion Criteria

We conducted a systematic review following the guidelines of the Cochrane Handbook 5.1 [13]. For this systematic review and meta-analysis, studies were considered eligible if they included any prophylactic interventions for healthy women with at least one BRCA mutation gene and collected data on outcomes such as incidence of gynecological cancers and cancer-related mortality.

To identify the research of prophylactic risk-management strategies in BRCA1/2 variant carriers, we then searched four databases, PubMed, Cochrane, Embase, and OVID Emcare, for research articles published after 1 January 2000. The key search terms included “BRCA mutation”, “BRCA variant”, “prophylactic intervention”, “chemoprevention”, “risk-reducing mastectomy”, “risk-reducing salpingo oophorectomy”, etc. We further limited our inclusion criteria to peer-reviewed published epidemiological, experimental, or observational studies, such as randomized controlled trials, cohorts, and case–control studies published in English; also, only human studies were considered and included. The detailed search term in each database has been listed in Supplementary Information Table S3. Qualitative studies, mathematical modeling studies, conference abstracts, reviews, letters erratum, and studies using self-reported outcomes were excluded from our analysis by screening titles and abstracts. Additionally, we further screened the full text of articles to exclude studies targeting participants with BRCA mutations who had a history of gynecologic-related cancer. The preliminary search originated on 20 August 2023, and the search was updated before the final analysis to include any recently published research on 13 October 2023. The PROSPERO registration number is 492969.

T.L., Y.J., Y.Y.G., and X.M. independently performed all of the stages of screening, and discrepancies in each stage of screening were discussed and resolved by the reviewers, T.L., Y.J., Y.Y.G., X.M., and S.J., and the results of screening were checked again by S.J.

### 2.2. Data Extraction

For each included study, the four authors (T.L., Y.J., Y.Y.G., X.M.) extracted the information from the following dimensions based on an extraction form developed by the senior author (S.J.). The form was initially developed according to the requirements by PRISMA and was later revised and confirmed through discussions among all authors. All retrieved information was examined by the three senior authors (S.J., W.K.M., and Y.G.). Any disagreements were resolved through discussions involving all the authors.

The extracted information included (1) study characteristics, including authors, study country, the aim of the study, and the year of publication; (2) methodological characteristics, including study participants (BRCA 1, BRCA2, or BRCA1/2 carriers), participant sample size, intervention types (RRS, chemoprevention, screening, genetic testing), and number of intervention mentioned in the study; and (3) outcome measures and value, including breast cancer and ovarian cancer incidence in the intervention and control groups, breast cancer-specific and ovarian cancer-specific mortality risk, and all-cause mortality during the follow-up period. In case the unadjusted data and adjusted data both mentioned the study, we only extract the adjusted data. The time of the follow-up period was also extracted.

### 2.3. Risk of Bias Assessment

We employed a modified version of the Newcastle–Ottawa Scale (NOS) to evaluate the methodological quality of each included study since most of our included studies were case–control studies and cohort studies [14]. The risk of bias assessments were independently carried out by three reviewers (T.L., J.Y., and Y.Y.G.). Discrepancies in the quality assessment step were resolved through discussions with the senior authors (S.J., W.K.M, and Y.G.). In the NOS criteria, studies receive a maximum of nine stars, with a maximum of four stars in Selection (four items and a maximum of one per item), two stars in Comparability, and three stars in Outcome or Exposure (three items, one per item). Studies were defined as having a low risk of bias if they scored four stars in Selection, two stars in Comparability, and three stars in Outcome or Exposure. Studies with a medium risk of bias were defined as having only two or three stars in Selection, one in Comparability, and two in Exposure or Outcome. Studies with only one in Selection, one in Comparability, or zero stars in any of the three domains were defined to be at high risk of bias [14]. Publication bias was also evaluated using funnel plots by T.L., J.Y., and Y.Y.G.

### 2.4. Data Synthesis and Analysis

We used descriptive analysis to summarize study characteristics, such as study location, study year, and study types. In addition to using descriptive analysis, narrative synthesis was also applied to tabulate the included studies, focusing on authors, study location and study duration, study design, analysis, and outcome measures. For quantitative data synthesis, we compared the outcome of included studies and re-categorized the outcome into breast cancer-specific and ovarian cancer-specific incidence and breast cancer-specific and ovarian cancer-specific mortality.

Given the varied outcome measures across the studies, we undertook a transform approach to ensure cross-study comparability. For metrics sharing similar interpretations among the included studies, such as risk ratios, were transformed to odds ratios (OR,), while the mortality rate was transformed to a hazard ratio (HR). The requisite raw data were extracted by T.L. and J.Y. and subsequently used to derive ORs and HRs employing contingency tables. In addition, the included studies were deemed to be meta-analyzed; specifically, the OR and HR directly extracted from the included studies were meta-analyzed, and self-calculated data using the sample size in the intervention and control groups of included studies were also meta-analyzed. The results of the extracted data analysis and self-calculated data analysis were reported separately and compared.

Due to the heterogeneity in studies regarding year, geographical context, and confounders, the 95% confidence intervals (CIs) derived from primary data and back-transform data were pooled using a random effects model. The between-study variance was calculated utilizing the DerSimonian–Laird estimator. To quantify the magnitude of inter-study variability, heterogeneity across the estimates was assessed using Higgins’ $I^{2}$ statistic, which evaluates the proportion of observed variance indicative of true effect sizes. An $I^{2}$ static of 50% or greater was deemed indicative of significant inter-study heterogeneity.

After the baseline meta-analysis for the estimation of the OR and HR, we also performed a subgroup analysis to provide more clinical insights. The subgroup analysis was conducted according to the OR and HR of the BRCA variant types (i.e., BRCA1, BRCA2, BRCA1/2), intervention types (i.e., RRM, RRSO, chemoprevention), and outcome of disease (i.e., breast cancer, ovarian cancer, other gynecological cancer).

Analyses were conducted using the “metan” package in STATA (version 18.0, StataCorp LLC, College Station, TX, USA), and we reported our results and findings according to Preferred Reporting Items for Systematic Reviews and Meta-Analysis (PRISMA) guidelines [15].

## 3. Results

### 3.1. Narrative Synthesis

**Figure 1:**
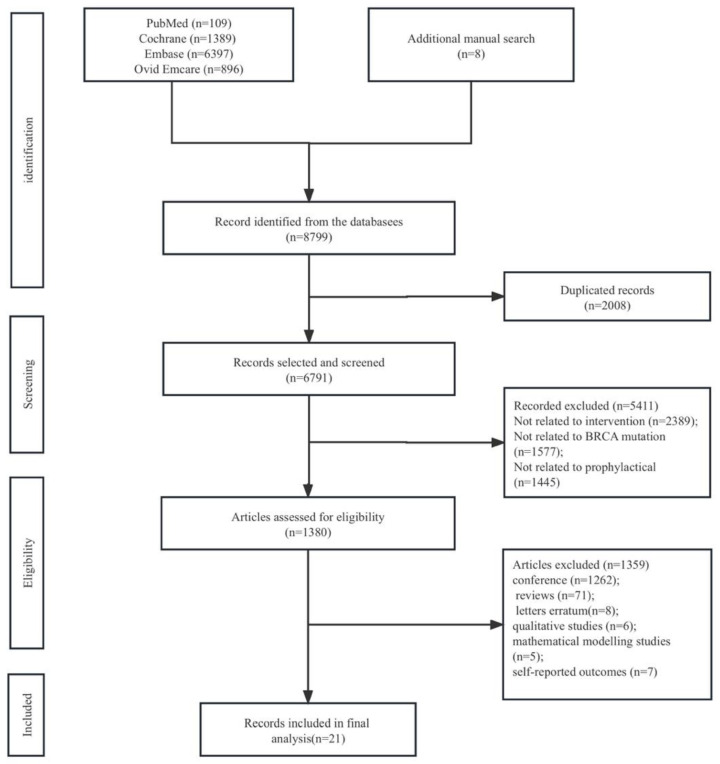
Flowchart of the search results.

Our search identified 8799 studies: 109 from PubMed, 1389 from Cochrane, 6397 from Embase, and 896 from Ovid Emcare, and there were eight records identified from a manual search of Google Scholar, other review articles, and reference lists of searched records. After excluding 2008 duplicate records, a total of 6791 records were included in the screening process. A total of 5411 records that are not related to intervention, BRCA mutation, or prophylactic interventions for BRCA carriers were excluded, leading to 1380 studies for further eligibility screening. Study types were further limited to only include epidemiologic studies, RCT, case–control studies, cohort studies, etc., and 1359 studies were excluded. A total of 21 articles were included in the data synthesis analysis (Figure 1). The characteristics of the included articles are summarized in Table 1.

The narrative synthesis of the distribution of the study location, publication year, study purpose, methodological characteristics, and outcome measures have been summarized in Table 1. Around half of the included studies (ten out of the twenty-one studies) were performed after 2010, and four studies were published after 2015. Ten studies were conducted in multi-centers across several countries, and these included studies that provided estimates predominantly in North America (e.g., the US and Canada), Europe (e.g., Netherlands, Denmark, Poland, France, Italy, Norway), and Israel. Also, sixteen studies were prospective cohort studies, four studies were case–control studies, and only one study was an RCT. The participants in all included studies were women BRCA1/2 variant carriers, prospective cohort studies were healthy women carriers, and case–control studies were patient carriers and control carriers. Some studies limited the age of participants to 30 [25] or 35 years old [26], and one study had an additional participant inclusion criteria: individuals who were BRCA1/2 carriers with a 1.66% risk of BC over the next 5 years [26]. Twelve studies covered oophorectomy and seven studies covered mastectomy, and Domchek et al. (2010) [10] covered oophorectomy and mastectomy simultaneously. Two studies discussed the effectiveness of chemoprevention (i.e., tamoxifen). One study, Kotsopoulos et al. (2005) [27], demonstrated the association between body weight change in BRCA1/2 variant carriers and the incidence of breast cancer and ovarian cancer. Moreover, six studies focused on the effectiveness of intervention in reducing mortality risk, nineteen studies investigated whether interventions reduce cancer risk, and four studies mentioned both cancer risk and mortality risk reduction. Four studies, specifically, Kauff et al. (2008) [25], Rebbeck et al. (2002) [31], Rebbeck et al. (2004) [32], Domchek et al. (2006) [16], and Domchek et al. (2010) [10], shared the same PROSE consortium datasets.

### 3.2. Meta-Analysis Results

**Figure 2:**
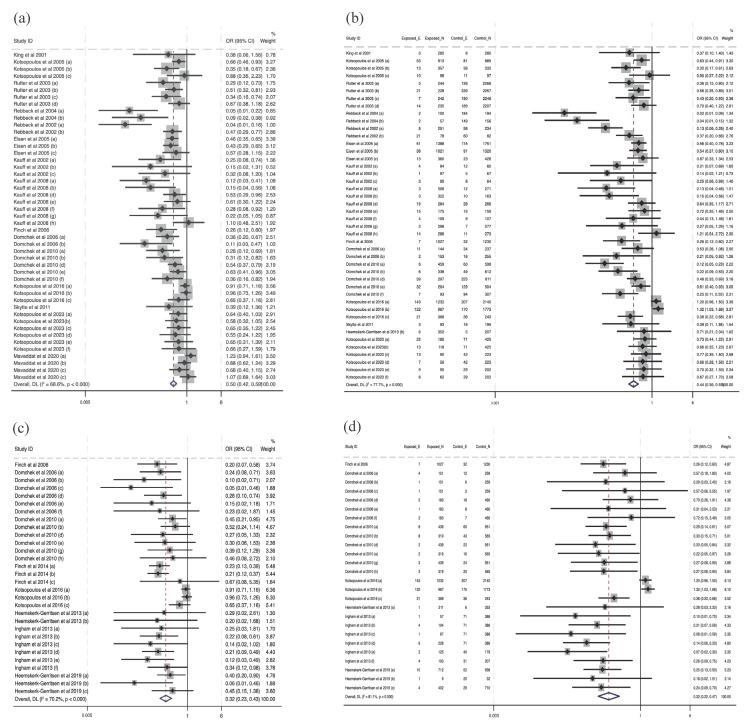
Forest plots of intervention effectiveness for cancer risk and mortality risk reduction [10,12,16,17,18,19,20,21,22,24,25,26,27,28,29,31,32,33,34]. Notes: panel (**a**) provides the synthesis of odds ratios for cancer risk reduction with data directly extracted from the included studies, and panel (**b**) provides the synthesis of odds ratios for cancer risk reduction with self-calculated data. Panels (**c**,**d**) provide a synthesis of odds ratios for mortality risk reduction, with panel (**c**) using direct extraction data and panel (**d**) using self-calculated data.

All the studies have been included in the meta-analysis. The clarification of sub-studies in Figures are mentioned in Tables S1 and S2. In the overall comparisons, the effectiveness of interventions mentioned in the studies indicated a significant reduction in cancer risk at post-intervention compared with the control conditions using data directly extracted from the studies (OR = 0.48, 95% CI (0.41, 0.56), $I^{2}$ = 68.0%, *p* < 0.001), and self-calculated data (OR = 0.44, 95% CI (0.36, 0.54), $I^{2}$ = 77.2%, *p* < 0.001). Also, compared with the effectiveness for cancer risk reduction, there was a larger protective effect on the mortality of interventions, both in the data directly extracted from studies (OR = 0.32, 95% CI (0.24, 0.44), $I^{2}$ = 70.2%, *p* < 0.001) and in the self-calculated data (OR = 0.32, 95% CI (0.22, 0.48), $I^{2}$ = 80.1%, *p* < 0.001). Substudies from studies conducted by Rebbeck et al., 2004 [32], Kauff et al., 2008 [25], Domchek et al., 2010 [10], Heemskerk-Gerritsen, 2013 and 2019 [20,21], Kaas et al., 2010 [23], and Meijers-Heijboer et al., 2001 [30], as well as Finch et al., 2014 [19], were excluded from Figure 2b–d, and substudies by Mavaddat et al., 2020 [29] were omitted from Figure 2a since there were no cancer cases or deaths in the intervention group, so the estimated ORs were zero and thus the 95% CI cannot be estimated. The analysis identified several outliers in the substudies. Specifically, Kauff et al., 2008 [25] (OR = 1.10, 95% CI (0.48, 2.51)) and Mavaddat et al., 2020 [29] (OR = 1.23, 95% CI (0.94, 1.61)), as shown in Figure 2a, and Kauff et al., 2008 [25] (OR = 1.21, 95% CI (0.54, 2.72)) and Kotsopoulos et al., 2017 [28] in Figure 2b–d, reported findings where the intervention condition was associated with an increased cancer risk or mortality risk. 

### 3.3. Subgroup Analysis

**Figure 3:**
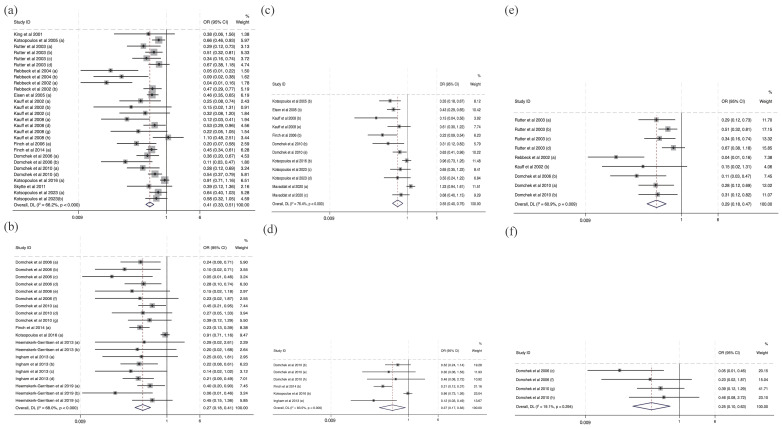
Forest plots of subgroup analysis for intervention for BRCA1/2, BRCA1, and BRCA2 mutation carriers [10,12,16,17,18,19,20,21,22,24,25,26,27,28,29,31,32,33,34]. Notes: panel (**a**) provides a synthesis of odds ratios for cancer risk reduction in all BRCA mutations. Panel (**b**) displays odds ratios for disease risk associated with BRCA1 mutations. Panel (**c**) presents odds ratios for disease risk linked to BRCA2 mutations. Panels (**d**–**f**) illustrate the synthesis of ORs for mortality risk reduction corresponding to all BRCA mutations, BRCA1 mutations, and BRCA2 mutations, respectively.

All the results presented for subgroup analysis were data directly extracted from included studies, and figures of self-calculated data are presented in Supplementary Information Tables S1 and S2 and Figures S2–S5. Figure 3 summarizes the subgroup analysis for the effectiveness of interventions in cancer risk and mortality risk reduction specific to BRCA1/2, BRCA1, and BRCA2 variant carriers. Fourteen studies discussing cancer risk and seven studies discussing mortality risk were included in this subgroup analysis. Consistent with Figure 2, interventions statistically significantly reduce cancer risk (OR = 0.41, 95% CI (0.33, 0.51)) and mortality risk (OR = 0.27, 95% CI (0.18, 0.41)) with BRCA1/2 variant carriers. In Figure 3c,e, interventions for cancer risk for BRCA2 variant carriers (OR = 0.29, 95% CI (0.18, 0.47)) have a greater protective effect than BRCA1 variant carriers (OR = 0.55, 95% CI (0.40, 0.76)). Also, in Figure 3d,f, interventions reduce more mortality risk in BRCA 2 (OR = 0.25, 95% CI (0.10, 0.63)) variant carriers than BRCA 1 variant carriers (OR = 0.37, 95% CI (0.17, 0.83)).

**Figure 4:**
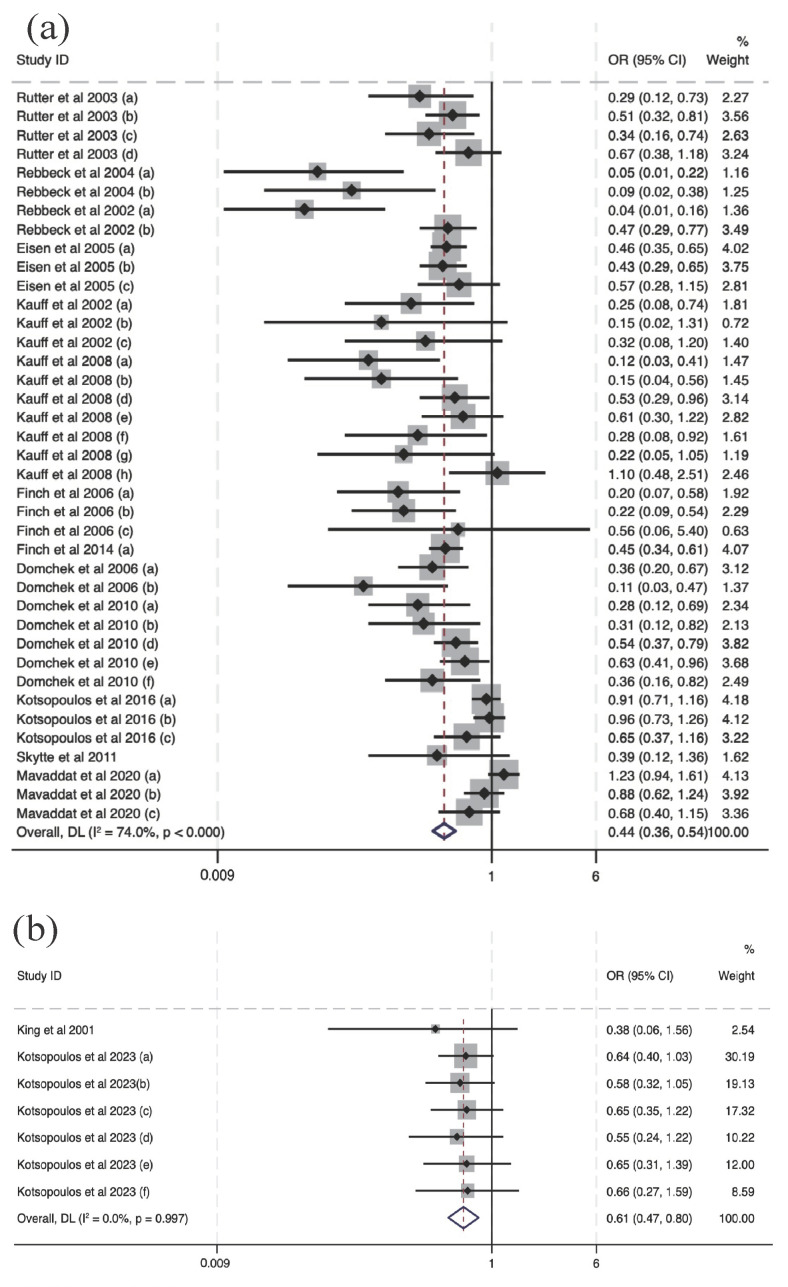
Forest plots of subgroup analysis for the effectiveness of RRS and chemoprevention [10,12,16,17,18,24,25,26,28,29,31,32,33,34]. Notes: panel (**a**) presents the effectiveness of RRS for cancer risk and panel (**b**) presents the effectiveness of chemoprevention for cancer risk.

There was a greater protective effect of RRS (OR = 0.44, 95% CI (0.36, 0.54)) than the chemoprevention of tamoxifen (OR = 0.61, 95% CI (0.47, 0.80), Figure 4). There was no analysis of intervention effectiveness for mortality risk with respect to RRS and chemoprevention since there were no discussions about mortality risk reduction in the two chemoprevention studies.

**Figure 5:**
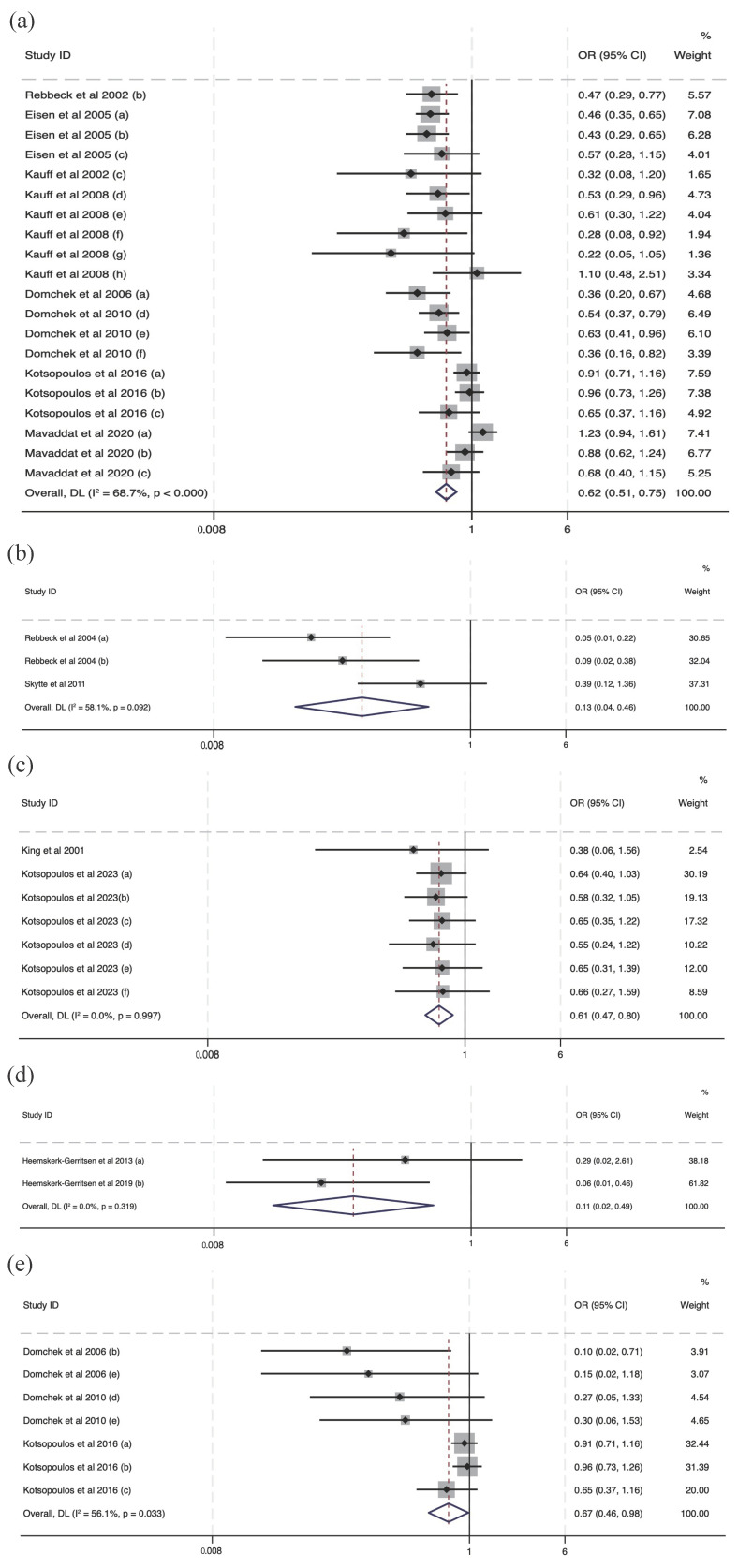
Forest plots of different interventions’ effectiveness in BC risk and mortality [10,12,16,17,20,21,24,25,26,28,29,32,34]. Notes: panel (**a**) indicates the effectiveness of oophorectomy in BC risk reduction. Panel (**b**) indicates the effectiveness of mastectomy in BC risk reduction. Panel (**c**) indicates the effectiveness of chemoprevention in BC risk reduction. Panel (**d**) indicates the effectiveness of mastectomy in BC-specific mortality risk reduction. Panel (**e**) indicates the effectiveness of oophorectomy in BC-specific mortality risk reduction.

Regarding the subgroup analysis of interventions related to specific cancer types, we observed that prophylactic oophorectomy (PO) significantly reduces the risk of breast cancer (BC) (OR = 0.62, 95% CI (0.51, 0.75), *p* < 0.001, Figure 5a) and BC-specific mortality (OR = 0.67, 95% CI (0.46, 0.98), *p* < 0.05, Figure 5e). Similarly, prophylactic mastectomy demonstrates a significant reduction in BC-specific risk (OR = 0.13, 95% CI (0.04, 0.46), *p* < 0.1, Figure 5b), although the reduction in BC-specific mortality did not achieve statistical significance (OR = 0.11, 95% CI (0.02, 0.49), Figure 5d). The effects of chemoprevention, such as tamoxifen, did not show a significant association with a reduction in BC-specific risk (OR = 0.61, 95% CI (0.47, 0.80), Figure 5c). Therefore, in terms of the prevention of breast cancer, prophylactic mastectomy presented better outcomes than prophylactic oophorectomy and chemoprevention.

**Figure 6:**
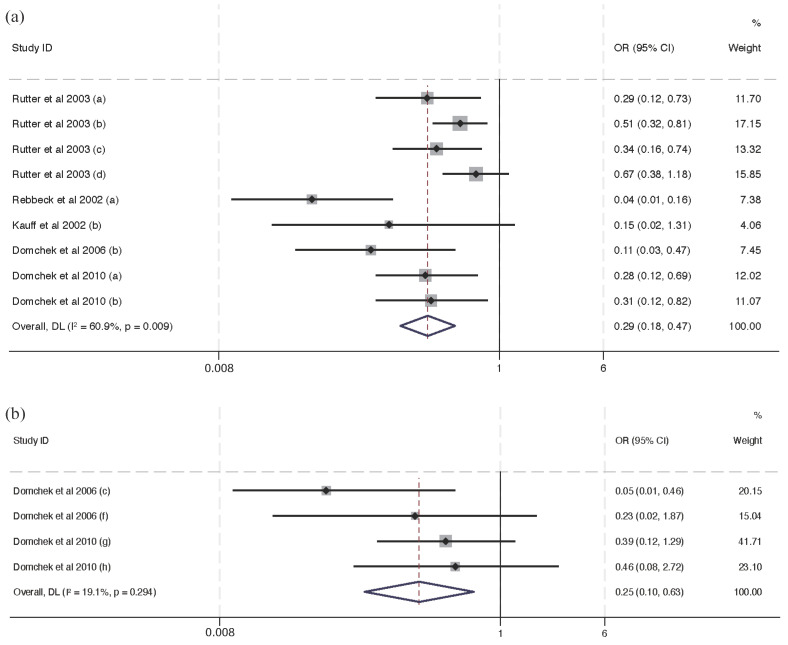
Forest plots of the effectiveness of interventions in OC risk and mortality risk reduction [10,16,24,31,33]. Notes: panel (**a**) indicates the effectiveness of oophorectomy in OC risk reduction and panel (**b**) indicates the effectiveness of oophorectomy in OC-specific mortality risk reduction.

Prophylactic oophorectomy reached a statistically significant level and was found to have a protective effect on OC risk (OR = 0.29, 95% CI (0.18, 0.47), *p* < 0.01, Figure 6a), and there was no statistically significant effect on OC-specific mortality (Figure 6b) (OR = 0.62, 95% CI (0.51, 0.75)).

### 3.4. Quality Assessment

The assessment of risk of bias is shown in Table 2. Since the study by King et al., 2001 [26] was an RCT study, it has been omitted in the NOS assessment, and 20 studies have been included in the NOS assessment. The case–control studies were assessed based on the NOS coding manual for case–control studies, and cohort studies were based on the NOS coding manual for cohort studies [14]. The risk of bias in the studies was higher if the score of the NOS was lower. The average mean score of the studies was 7.75, with seven studies scoring a maximum of nine and one study scoring a minimum of four. Additionally, three studies were considered as having a high risk of bias due to their one-star score in the Exposure/Outcome aspect of the NOS. The publication bias of the included studies is summarized in Supplementary Information Figure S1.

**Figure 7:**
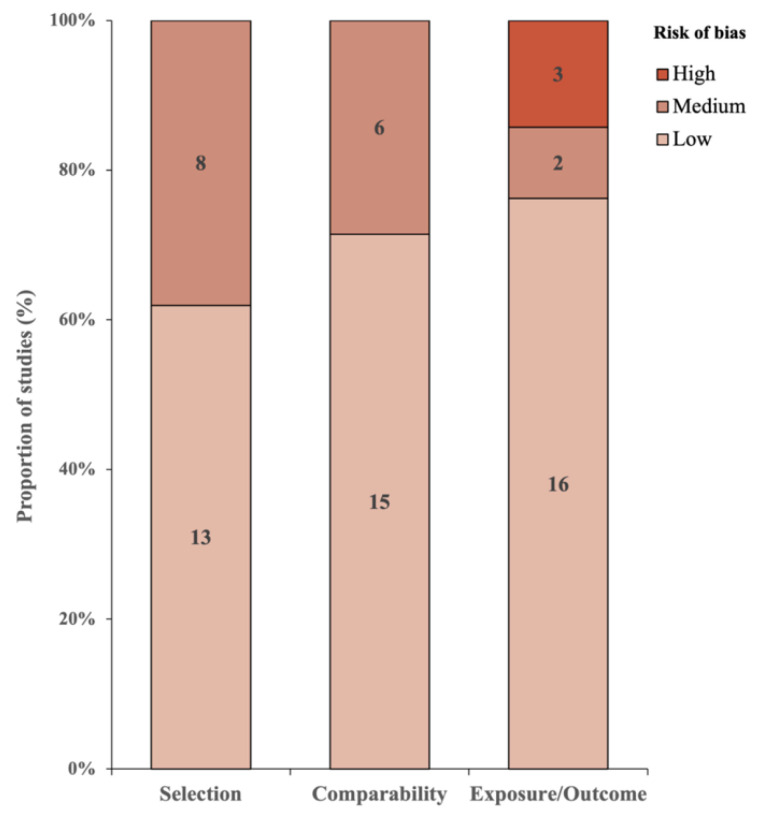
Distribution of the risk of bias across the studies.

Figure 7 illustrates the risk of bias across three categories: Selection, Comparability, and Exposure/Outcome. For each category, the studies are divided into three levels of bias: high, medium, and low. In the Selection category, eight studies exhibited a high risk of bias, while thirteen displayed a low risk. Comparability had six studies with a high risk of bias and fifteen with a low risk of bias. Lastly, in the Exposure/Outcome category, three studies had a high risk of bias, two had a medium risk of bias, and sixteen had a low risk of bias.

## 4. Discussion

We conducted a systematic review and meta-analysis of the current prophylactic interventions and their effectiveness in mitigating the risk of hereditary breast and ovarian cancers among carriers of BRCA1 or BRCA2 mutations. Our study suggested that, overall, prophylactic HBOC interventions are strongly associated with a significant reduction in cancer risk and mortality risk. This finding was consistent with the previous literature. Moreover, the present study discussed the effectiveness of various prophylactic interventions from both cancer risk and mortality aspects, which has not been discussed thoroughly in the previous studies; therefore, we clinically highlighted the significance of the prophylactic interventions for BRCA1/2 variant carriers’ quality of life improvement and life years extension in the long term [35].

Heterogeneities of interventions’ effectiveness were also found in our subgroup analysis in carriers with different BRCA mutations. As was mentioned in the previous studies [36,37], the risk for malignancy in BRCA1 carriers was found to be 55–72% by age 70, much higher than 45–69% for BRCA2 carriers. Moreover, in our study, we observed a notable difference in the effectiveness of risk-reduction prophylactic interventions between BRCA1 and BRCA2 variant carriers. Specifically, BRCA1 variant carriers received a comparatively lower protection effect than BRCA2 carriers. This highlighted the requirements for BRCA1 carriers to have more precision and earlier prophylactic interventions. These may include more intensive screening strategies, e.g., the combination of mammography and MRI [38] and genetic counseling, which may help detect cancer’s onset at an earlier stage, delay the disease’s progression, and enhance the quality of life for BRCA1 variant carriers. It is worth noting that our findings align with the current National Comprehensive Cancer Network (NCCN) guidelines, in which the recommended age for RRBSO is younger for BRCA1 carriers (35–40 years) compared to BRCA2 carriers (40–45 years) [39].

The results of the comparison between RRS and chemoprevention revealed that the overall effectiveness of surgery is higher than chemoprevention, indicating that surgery is still the most effective prophylactic strategy for HBOC [40]. Specifically, the RRS strategies reduced cancer risk by 56%, and chemoprevention provided a 39% reduction in cancer risk. However, the effectiveness of chemoprevention does not reach a statistically significant level, which indicates that the effect of using chemoprevention as a prophylactic strategy remains unclear, which may be due to the very few studies (n = 2) included in this study. This finding was also consistent with the previous studies [41]. Chemoprevention may or may not be clinically effective for cancer prevention, which warrants more studies to confirm this conclusion to support the medical decision-making process.

In the twenty-one included studies in our study, there were three main prophylactic strategies for BC risk, specifically RRM, risk-reducing oophorectomy (RRO), and chemoprevention. We found that RRM resulted in an 87% reduction in BC risk (*p* < 0.1), although it did not significantly alter mortality risk. In contrast, RRO demonstrated a significant decrease in both BC risk and mortality risk compared to RRM. Despite these findings, some studies presented contrasting results; for instance, Kauff et al. (2008) [25] and Mavaddat et al. (2020) [29] reported that RRO did not significantly reduce the risk of ER-negative BC and BC risk in BRCA1 carriers, respectively. This is in line with Kotsopoulos et al. (2017) [28], who indicated a protective effect of oophorectomy only in BRCA2 variant carriers. This discrepancy can be logically explained by considering the relationship between BRCA1 mutations and ER-negative BC. Specifically, breast cancer tends to develop earlier in BRCA1 variant carriers, and their tumors are predominantly ER-negative. The early onset of cancer suggests that the factors causing cancer in BRCA1 carriers might manifest before many women undergo RRO, influencing disease incidence. In contrast, BRCA2-associated tumors are mostly ER-positive, and early menopause, potentially induced by RRO, is associated with a reduced risk of ER-positive BC. Thus, understanding these nuances in the relationship between BRCA1 and ER-negative BC necessitates further research focused on the molecular subtypes of breast cancer.

Compared with various prophylactic strategies for BC risk and mortality risk reduction, we found only one prophylactic strategy for OC-specific risk reduction, i.e., oophorectomy. PO reduced around 71% of OC risk (*p* < 0.01), and although PO provides 38% risk-reduction effectiveness in OC-specific mortality, such a protective effect on OC-specific mortality was not statistically significant. This indicates that currently, the strategies for OC risk reduction are very limited, and the evidence for OC-specific mortality remains insufficient. Several studies have suggested that prophylactic salpingectomy is also effective in preventing ovarian cancer. This strategy is based on the theory that most hereditary epithelial ovarian cancers originate in the fallopian tubes [42,43]. However, there are currently no clinical studies (e.g., RCTs and observational studies) to support this theory, and future studies are needed to enhance this area of research to provide more prophylactic and strategic options for OC-specific cancer and mortality risk reduction [43].

The current literature neglects the side effects and downstream consequences of RRS and chemoprevention from either a clinical perspective or a societal perspective. Clinically, RRS may result in decreased cognition and sexual function and an increased risk of osteoporosis and cardiac mortality [44,45], and chemoprevention may lead to menopausal-related symptoms, blood clots, stroke, or an increased risk of endometrial cancer and hormone disorders [44]. In addition, a study by Shu, C. A et al. discussed that undergoing RRSO may increase the risk for serous/serous-like endometrial carcinoma in BRCA1 variant carriers [46]. Preventing breast and ovarian cancers could yield savings in publicly funded insurance schemes, yet the associated expenses from managing side effects and consequences of risk-reducing surgery (RRS) and chemoprevention raise concerns. The net cost effectiveness of these strategies, when considering the full scope of medical costs—both directly and indirectly related to treatment—as well as their impact on economic productivity, remains uncertain [47,48]. This ambiguity arises due to the absence of comprehensive economic models that encapsulate all resultant consequences and associated costs [49]. In addressing this gap, the consideration of methodologies, such as whole disease modeling, could prove instrumental [50].

Beyond RRS and chemoprevention, lifestyle modification emerges as a viable strategy for mitigating the risk of breast cancer. Notably, BRCA variant carriers who experienced a weight loss of at least 10 pounds between the ages of 18 and 30 exhibited a 34% reduction in breast cancer risk [27]. Therefore, lifestyle modification could be posited as an alternative strategy for breast cancer risk reduction. However, the evidence to substantiate this is not yet robust enough for meta-analysis. Moreover, the impact of lifestyle modification on reducing ovarian cancer risk remains undetermined, underscoring the need for additional real-world evidence to ascertain the clinical effectiveness of lifestyle modification in reducing the risk of breast and ovarian cancers.

The cost effectiveness of the preventive strategies is contingent not only on their clinical effectiveness but also the extent of their uptake. An intervention cannot achieve its cost effectiveness when no patients use it, assuming that a complete uptake (i.e., a 100% uptake) among patients is impractical due to the inherent preference heterogeneity and the emphasis on patient-centered care in oncological contexts [51]. Hence, when devising economic models to assess the cost effectiveness of these preventive strategies, uptake rates constitute crucial parameters. It is worth noting that uptake rates exhibit significant variability across different countries and regions. For instance, in the case of BRCA1/2 carriers, a preference for chemoprevention was observed in the US (12.4%, tamoxifen and raloxifene), whereas the uptake rate for chemoprevention was nearly negligible among carriers from European countries like Norway, Italy, and the Netherlands [52]. The uptake rates of RRS also differed, e.g., 27% in the US, 65% in Spain, and 88% in the UK [53]. Therefore, when establishing economic models, it is imperative to ensure that the uptake parameters are context-specific to avoid under- or over-estimating the cost effectiveness of the prophylactic interventions [49].

Another issue regarding uptake rates is the predominant focus on evidence from developed nations. There is limited evidence from developing countries, where the guidelines for HBOC risk management, prophylactic strategies, genetic testing, and counselling are lacking [54]. The extrapolation of uptake parameters from developed to developing countries could lead to an inaccurate estimation of the cost effectiveness of interventions. For example, should the BRCA variant carriers in developing countries exhibit a higher propensity to opt for RRS compared to their counterparts in developed countries, the cost effectiveness of RRS could significantly diverge. This observation, aligned with the existing literature, accentuates the necessity for further studies grounded in developing countries, aiding the decision-makers in these countries to formulate judicious and economically feasible recommendations for both the healthcare system and society at large.

## 5. Conclusions

Prophylactic interventions for healthy BRCA1/2 variant carriers are associated with a reduction in HBOC cancer and mortality risk. In contrast, chemoprevention does not significantly alter these risks. Heterogeneity in the efficacy among different prophylactic approaches, cancer types, and genetic mutations is evident. Our systematic review contributes substantial evidence that is instrumental for future economic evaluation of HBOC interventions across diverse international contexts.

## Figures and Tables

**Table 1:** Narrative synthesis of the selected studies.

Study	Study Country	Study Purpose	Study Design	Participants	Sample Size	Intervention	Outcomes
Domchek et al. (2006) [16]	US and Europe	Estimating overall mortality or BRCA-related gynecologic cancer-specific mortality reduction after BPSO	Prospective cohort study	Women with BRCA1 or BRCA2 mutations	426 for primary analysis, 666 for secondary analysis	BPSO	HR for cancer risk and mortality
Domchek et al. (2010) [10]	North America and Europe	Estimating BC- and OC-specific risk and mortality reduction after RRM and RRSO	Prospective cohort study	Women with BRCA1 or BRCA2 mutations	2482	RRM. RRSO	HR for cancer risk and mortality
Eisen et al. (2005) [17]	North America, Europe, and Israel	Estimating BC-specific risk reduction after PO	Case–control study	Patients with breast cancer and matched controls with BRCA mutations	1439 patients, 1866 controls	PO	OR for cancer risk
Finch et al. (2006) [18]	Canada, US, Europe, and Israel	Estimating OC-and other BRCA-related gynecologic cancer-specific risk reduction after BPSO	Prospective cohort study	Women with BRCA1 or BRCA2 mutations	1828	BPSO	Incidence rate and HR for cancer risk
Finch et al. (2014) [19]	Canada, US, Austria, France, Italy, Norway, Poland	Estimating OC- and other BRCA-related gynecologic cancer-specific risk and all-cause mortality reduction after PO	Prospective cohort study	Women with BRCA1 or BRCA2 mutations	5783	PO	HR for cancer risk and mortality
Heemskerk- Gerritsen et al. (2013) [20]	Netherlands	Estimating BC-specific risk and mortality reduction after BRRM	Prospective cohort study	Women with BRCA1 or BRCA2 mutations	570	BRRM	Incidence rate for cancer risk and HR for mortality
Heemskerk- Gerritsen et al. (2019) [21]	Netherlands	Estimating BC-specific mortality reduction after BRRM	Prospective cohort study	Women with BRCA1 or BRCA2 mutations	2857	BRRM	HR for mortality
Ingham et al. (2013) [22]	UK	Estimating BC- and OC-specific mortality reduction after BRRM and BRRSO	Prospective cohort study	Women with BRCA1 or BRCA2 mutations	691	BRRSO	HR for mortality
Kaas et al. (2010) [23]	Netherlands	Estimating BC-specific risk reduction after BRRM	Prospective cohort study	Women with BRCA1 or BRCA2 mutations	254	BRRM	Incidence rate for cancer risk
Kauff et al. (2002) [24]	US	Estimating BC- and OC- and other BRCA-related gynecologic cancer-specific risk reduction after RRSO	Prospective cohort study	Women with BRCA1 or BRCA2 mutations, 35 years of age or older	265	RRSO	HR for cancer risk
Kauff et al. (2008) [25]	US	Estimating BC- and other BRCA-related gynecologic cancer-specific risk reduction after RRSO	Prospective cohort study	Women with BRCA1 or BRCA2 mutations, 30 years of age or older	1079	RRSO	HR for cancer risk
King et al. (2001) [26]	US	Estimating BC-specific risk reduction after chemoprevention of tamoxifen	Randomized controlled trial	Women with BRCA1 or BRCA2 mutations, 35 years of age or older with a 1.66% risk of BC over the next 5 years	13,195	Tamoxifen	RR for cancer risk
Kotsopoulos et al. (2005) [27]	Canada, Israel, UK, Poland, US	Estimating BC-specific risk reduction after a change in body weight	Case–control study	Women with BRCA1 or BRCA2 mutations	1073 patients, 1073 controls	Body weight change	OR for cancer risk
Kotsopoulos et al. (2017) [28]	12 countries including Canada and the US	Estimating BC-specific risk reduction after BO	Prospective cohort study	Women with BRCA1 or BRCA2 mutations	3722	BO	HR and adjusted HR for cancer risk
Kotsopoulos et al. (2023) [12]	17 countries including Canada and the US	Estimating BC-specific risk reduction after chemoprevention of tamoxifen	Prospective cohort study	Women with BRCA1 or BRCA2 mutations	4578	Tamoxifen	HR for cancer risk
Mavaddat et al. (2020) [29]	UK	Estimating BC-specific risk reduction after RRSO	Prospective cohort study	Women with BRCA1 or BRCA2 mutations	3877	RRSO	HR for cancer risk
Meijers- Heijboer et al. (2001) [30]	Netherlands	Estimating BC-specific risk reduction after BRRM	Prospective cohort study	Women with BRCA1 or BRCA2 mutations	139	BRRM	HR for cancer risk
Rebbeck et al. (2002) [31]	North America, Europe	Estimating BC- and OC-specific risk reduction after PO	Prospective cohort study	Women with BRCA1 or BRCA2 mutations	551	PO	HR for cancer risk
Rebbeck et al. (2004) [32]	North America, Europe	Estimating BC-specific risk reduction after BPM	Case–control study	Women with BRCA1 or BRCA2 mutations	105 patients, 378 controls	BPM	Adjusted HR for cancer risk
Rutter et al. (2003) [33]	Israel	Estimating OC-specific risk reduction after BRCA-related gynecologic surgeries	Case–control study	Jewish women confirmed with epithelial ovarian cancer or primary peritoneal cancer	1124 patients, 2396 controls	BO, gynecologic surgeries with or without ovarian tissue removed	OR for cancer risk
Skytte et al. (2011) [34]	Denmark	Estimating BC-specific risk reduction after BRRM	Prospective cohort study	Women with BRCA1 or BRCA2 mutations	307	BRRM	Incidence rate for cancer risk

Notes: BC: breast cancer, OC: ovarian cancer, RRM: risk-reducing mastectomy, BPSO: bilateral prophylactic salpingo-oophorectomy, PO: prophylactic oophorectomy, BRRM: bilateral risk-reducing mastectomy, BRRSO: bilateral risk-reducing salpingo-oophorectomy, BO: bilateral oophorectomy, BPM: bilateral prophylactic mastectomy, RR: risk ratio, HR: hazard ratio, OR: odds ratio.

**Table 2:** Risk of bias assessment using the Newcastle–Ottawa Scale (NOS).

Study and Publication Years	Selection				Comparability	Exposure/Outcome			Score
Domchek et al., 2006 [16]	*	*	*	*	**	*	*	*	9
Domchek et al., 2010 [10]	*	*	*		**	*	*	*	8
Eisen et al., 2005 [17]	*	*	*	*	*	*	*		7
Finch et al., 2006 [18]	*	*	*	*	**	*	*	*	9
Finch et al., 2014 [19]	*	*	*		*	*	*	*	7
Heemskerk-Gerritsen et al., 2013 [20]	*	*	*	*	**	*		*	8
Heemskerk-Gerritsen et al., 2019 [21]	*	*	*	*	**	*	*	*	9
Ingham et al., 2013 [22]		*	*	*	*	*	*	*	7
Kaas et al., 2010 [23]		*	*	*	*	*	*	*	7
Kauff et al., 2002 [24]		*	*		**	*	*	*	7
Kauff et al., 2008 [25]	*	*	*		**	*	*	*	8
Kotsopoulos et al., 2005 [27]	*	*	*	*	**		*		7
Kotsopoulos et al., 2017 [28]	*	*	*	*	**	*	*	*	9
Kotsopoulos et al., 2023 [12]	*	*	*	*	**		*		7
Mavaddat et al., 2020 [29]	*	*	*	*	*	*	*	*	8
Meijers-Heijboer et al., 2001 [30]		*	*		**	*	*	*	7
Rebbeck et al., 2002 [31]	*	*	*	*	**	*	*	*	9
Rebbeck et al., 2004 [32]	*	*	*	*	**	*	*	*	9
Rutter et al., 2003 [33]	*		*	*	*				4
Skytte et al., 2011 [34]	*	*	*	*	**	*	*	*	9

Notes: Studies were defined as having a low risk of bias if they scored four stars in Selection, two stars in Comparability, and three stars in Outcome or Exposure. Studies with a medium risk of bias were defined as having only two or three stars in Selection, one in Comparability, and two in Exposure or Outcome. Studies with only one in Selection, one in Comparability, or zero stars in any of the three domains were defined to be at high risk of bias.

## Data Availability

Data are available after the request for the Corresponding authors: Prof. Yuanyuan Gu (yuanyuan.gu@mq.edu.au) and Prof. Wai-kit Ming (wkming2@cityu.edu.hk).

## Supplementary Materials

The following supporting information can be downloaded at https://www.mdpi.com/article/10.3390/cancers16010103/s1, Table S1: Odds ratio for disease, Table S2: Odds ratio for mortality, Table S3: Search strategy, Figure S1: Funnel plot, Figure S2: Self-calculated forest plots of subgroup analysis for the interventions of BRCA1/2, BRCA1, and BRCA2 mutation carriers, Figure S3: Self-calculated forest plots of subgroup analysis for the effectiveness of RRS and chemoprevention, Figure S4: Self-calculated forest plots of different interventions’ effectiveness in BC-specific cancer risk and mortality, Figure S5: Self-calculated forest plots of the effectiveness of interventions in OC-specific cancer risk and mortality risk reduction. References [10,12,16,17,18,19,20,21,22,24,25,26,27,28,31,32,33,34] are cited in the supplementary materials.
