# Supplementary material for: Prophylactic Interventions for Hereditary Breast and Ovarian Cancer Risks and Mortality in BRCA1/2 Carriers
Source: Cancers (Basel). 2023 Dec 24;16(1):103. doi: 10.3390/cancers16010103 (PMC10778044; doi:10.3390/cancers16010103)
Supplement: Supplementary file 1 [file cancers-16-00103-s001.zip › cancers-2744426-supplementary.pdf]

Supplementary Information

Contents

Table S1: Odds ratio for disease 2

Table S2: Odds ratio for mortality 4

Table S3: Search Strategy 5

Figure S1: Funnel Plot 12

Figure S2: Self-calculate forest plots of subgroup analysis for intervention for BRCA1/2, BRCA1, and BRCA2 mutation carriers 13

Figure S3. Self-calculate forest plots of subgroup analysis for effectiveness of RRS and chemoprevention 14

Figure S4. Self-calculate forest plots of different interventions effectiveness in BC-specific cancer risk and mortality 15

Figure S5: Self-calculated forest plots of effectiveness of interventions in OC-specific cancer risk and mortality risk reduction 16

Table S1. Odds ratio for disease.

Study ID	Mutation	Disease	Intervention	Subanalysis
King et al 2001	BRCA All	Breast Cancer	Pharmarcy	-
Kotsopoulos et al 2005 (a)	BRCA All	Breast Cancer	Change body weight	-
Kotsopoulos et al 2005 (b)	BRCA 1	Breast Cancer	Change body weight	-
Kotsopoulos et al 2005 (c)	BRCA 2	Breast Cancer	Change body weight	-
Rutter et al 2003 (a)	BRCA All	Ovarian Cancer	Bilateral oophorectomy	-
Rutter et al 2003 (b)	BRCA All	Ovarian Cancer	Any gynecologic surgery	-
Rutter et al 2003 (c)	BRCA All	Ovarian Cancer	Surgery with ovarian tissue removed	-
Rutter et al 2003 (d)	BRCA All	Ovarian Cancer	Surgery with ovarian tissue removed	-
Rebbeck et al 2004 (a)	BRCA All	Breast Cancer	Bilateral Prophylactic Mastectomy	Analysis 1
Rebbeck et al 2004 (b)	BRCA All	Breast Cancer	Bilateral Prophylactic Mastectomy	Analysis 2
Rebbeck et al 2004 (c)	BRCA All	Breast Cancer	Bilateral Prophylactic Mastectomy	Analysis 3
Rebbeck et al 2004 (d)	BRCA All	Breast Cancer	Bilateral Prophylactic Mastectomy	Analysis 4
Rebbeck et al 2002 (a)	BRCA All	Ovarian Cancer	Oophorectomy	-
Rebbeck et al 2002 (b)	BRCA All	Breast Cancer	Oophorectomy	-
Eisen et al 2005 (a)	BRCA All	Breast Cancer	Bilateral Oophorectomy	-
Eisen et al 2005 (b)	BRCA 1	Breast Cancer	Bilateral Oophorectomy	-
Eisen et al 2005 (c)	BRCA 2	Breast Cancer	Bilateral Oophorectomy	-
Kauff et al 2002 (a)	BRCA All	All Cancer	Salpingo-Oophorectomy	-
Kauff et al 2002 (b)	BRCA All	Ovarian Cancer	Salpingo-Oophorectomy	-
Kauff et al 2002 (c)	BRCA All	Breast Cancer	Salpingo-Oophorectomy	-
Kauff et al 2008 (a)	BRCA All	All Cancer	Salpingo-Oophorectomy	-
Kauff et al 2008 (b)	BRCA 1	All Cancer	Salpingo-Oophorectomy	-
Kauff et al 2008 (c)	BRCA 2	All Cancer	Salpingo-Oophorectomy	-
Kauff et al 2008 (d)	BRCA All	Breast Cancer	Salpingo-Oophorectomy	-
Kauff et al 2008 (e)	BRCA 1	Breast Cancer	Salpingo-Oophorectomy	-
Kauff et al 2008 (f)	BRCA 2	Breast Cancer	Salpingo-Oophorectomy	-
Kauff et al 2008 (g)	BRCA All	Breast Cancer	Salpingo-Oophorectomy	-

Kauff et al 2008 (h)	BRCA All	Breast Cancer	Salpingo-Oophorectomy	-
Finch et al 2006 (a)	BRCA All	All Cancer	Bilateral salpingo-oophorectomy	-
Finch et al 2006 (b)	BRCA 1	All Cancer	Bilateral salpingo-oophorectomy	-
Finch et al 2006 (c)	BRCA 2	All Cancer	Bilateral salpingo-oophorectomy	-
Finch et al 2014 (a)	BRCA All	All Cancer	Salpingo-Oophorectomy	-
Domchek et al 2006 (a)	BRCA All	Breast Cancer	Bilateral salpingo-oophorectomy	-
Domchek et al 2006 (b)	BRCA All	Ovarian Cancer	Bilateral salpingo-oophorectomy	-
Domchek et al 2010 (a)	BRCA All	Ovarian Cancer	Salpingo-oophorectomy	-
Domchek et al 2010 (b)	BRCA 1	Ovarian Cancer	Salpingo-oophorectomy	-
Domchek et al 2010 (c)	BRCA 2	Ovarian Cancer	Salpingo-oophorectomy	-
Domchek et al 2010 (d)	BRCA All	Breast Cancer	Salpingo-oophorectomy	-
Domchek et al 2010 (e)	BRCA 1	Breast Cancer	Salpingo-oophorectomy	-
Domchek et al 2010 (f)	BRCA 2	Breast Cancer	Salpingo-oophorectomy	-
Kotsopoulos et al 2016 (a)	BRCA All	Breast Cancer	Bilateral salpingo-oophorectomy	-
Kotsopoulos et al 2016 (b)	BRCA 1	Breast Cancer	Bilateral salpingo-oophorectomy	-
Kotsopoulos et al 2016 (c)	BRCA 2	Breast Cancer	Bilateral salpingo-oophorectomy	-
Skytte et al 2011	BRCA All	Breast Cancer	Bilateral Prophylactic Mastectomy	-
Heemskerk-Gerritsen et al 2013 (a)	BRCA All	Breast Cancer	Bilateral Prophylactic Mastectomy	-
Heemskerk-Gerritsen et al 2013 (b)	BRCA All	Ovarian Cancer	Bilateral Prophylactic Mastectomy	-
Kaas et al 2010	BRCA 1	Breast Cancer	Bilateral Prophylactic Mastectomy	-
Kaas et al 2010	BRCA 2	Breast Cancer	Bilateral Prophylactic Mastectomy	-
Kotsopoulos et al 2023 (a)	BRCA All	Breast Cancer	Chemoprevention either	-
Kotsopoulos et al 2023(b)	BRCA All	Breast Cancer	Chemoprevention Tamoxifen any	-
Kotsopoulos et al 2023 (c)	BRCA 1	Breast Cancer	Chemoprevention either	-
Kotsopoulos et al 2023 (d)	BRCA 1	Breast Cancer	Chemoprevention Tamoxifen any	-

Kotsopoulos et al 2023 (e)	BRCA 2	Breast Cancer	Chemoprevention either	-
Kotsopoulos et al 2023 (f)	BRCA 2	Breast Cancer	Chemoprevention Tamoxifen any	-
Mavaddat et al 2020 (a)	BRCA 1	Breast Cancer	Bilateral salpingo-oophorectomy	-
Mavaddat et al 2020 (b)	BRCA 2	Breast Cancer	Bilateral salpingo-oophorectomy	-
Mavaddat et al 2020 (c)	BRCA 1	Breast Cancer	Bilateral salpingo-oophorectomy	-
Meijers-Heijboer et al 2001	BRCA All	Breast Cancer	Bilateral Prophylactic Mastectomy	-

Table S2. Odds ratio for mortality.

Study ID	Mutation	Disease	Intervention
Domchek et al 2006 (a)	BRCA All	All Cancer	Bilateral salpingo-oophorectomy
Domchek et al 2006 (b)	BRCA All	Breast Cancer	Bilateral salpingo-oophorectomy
Domchek et al 2006 (c)	BRCA All	Ovarian Cancer	Bilateral salpingo-oophorectomy
Domchek et al 2006 (d)	BRCA All	All Cancer	Bilateral salpingo-oophorectomy
Domchek et al 2006 (e)	BRCA All	Breast Cancer	Bilateral salpingo-oophorectomy
Domchek et al 2006 (f)	BRCA All	Ovarian Cancer	Bilateral salpingo-oophorectomy
Domchek et al 2010 (a)	BRCA All	All Cancer	Salpingo-oophorectomy
Domchek et al 2010 (b)	BRCA 1	All Cancer	Salpingo-oophorectomy
Domchek et al 2010 (c)	BRCA 2	All Cancer	Salpingo-oophorectomy
Domchek et al 2010 (d)	BRCA All	Breast Cancer	Salpingo-oophorectomy
Domchek et al 2010 (e)	BRCA 1	Breast Cancer	Salpingo-oophorectomy
Domchek et al 2010 (f)	BRCA 2	Breast Cancer	Salpingo-oophorectomy
Domchek et al 2010 (g)	BRCA All	Ovarian Cancer	Salpingo-oophorectomy
Domchek et al 2010 (h)	BRCA 1	Ovarian Cancer	Salpingo-oophorectomy
Domchek et al 2010 (i)	BRCA 2	Ovarian Cancer	Salpingo-oophorectomy
Finch et al 2014 (a)	BRCA All	All Cancer	Oophorectomy
Finch et al 2014 (b)	BRCA 1	All Cancer	Oophorectomy
Finch et al 2014 (c)	BRCA 2	All Cancer	Oophorectomy

Kotsopoulos et al 2016 (a)	BRCA All	Breast Cancer	Bilateral Oophorectomy
Kotsopoulos et al 2016 (b)	BRCA 1	Breast Cancer	Bilateral Oophorectomy
Kotsopoulos et al 2016 (c)	BRCA 2	Breast Cancer	Bilateral Oophorectomy
Heemskerk-Gerritsen et al 2013 (a)	BRCA All	Breast Cancer	Bilateral risk-reducing Mastectomy
Heemskerk-Gerritsen et al 2013 (b)	BRCA All	All Cancer	Bilateral risk-reducing Mastectomy
Ingham et al 2013 (a)	BRCA All	All Cancer	Bilateral risk-reducing Mastectomy
Ingham et al 2013 (b)	BRCA All	All Cancer	Bilateral Oophorectomy
Ingham et al 2013 (c)	BRCA All	All Cancer	Bilateral risk-reducing Mastectomy & Bilateral Oophorectomy
Ingham et al 2013 (d)	BRCA All	All Cancer	Any primary preventive surgery
Ingham et al 2013 (e)	BRCA 1	All Cancer	Any primary preventive surgery
Ingham et al 2013 (f)	BRCA 2	All Cancer	Any primary preventive surgery
Heemskerk-Gerritsen et al 2019 (a)	BRCA All	All Cancer	Bilateral Prophylactic Mastectomy
Heemskerk-Gerritsen et al 2019 (b)	BRCA All	Breast Cancer	Bilateral Prophylactic Mastectomy
Heemskerk-Gerritsen et al 2019 (c)	BRCA All	All Cancer	Bilateral Prophylactic Mastectomy
Heemskerk-Gerritsen et al 2019 (d)	BRCA All	Breast Cancer	Bilateral Prophylactic Mastectomy

Table S3. Search Strategy.

Database name	Embase	Number
Date searched	Search conducted 13/10/23	6397
Search Strategy	hereditary breast cancer':ti,ab,kw OR 'hereditary breast cancer syndrome':ti,ab,kw OR 'hboc syndrome':ti,ab,kw OR 'brca':ti,ab,kw OR 'brca1 carrier':ti,ab,kw OR 'brca2 carrier':ti,ab,kw OR 'hereditary breast-ovarian cancer syndrome':ti,ab,kw OR 'hereditary breast-ovarian cancer':ti,ab,kw OR 'hereditary breast and ovarian cancer syndrome':ti,ab,kw OR 'hereditary breast and ovarian cancer':ti,ab,kw OR 'genetic variation breast cancer':ti,ab,kw OR 'breast cancer mutation':ti,ab,kw OR 'brca mutation':ti,ab,kw OR 'genetic variation ovarian cancer':ti,ab,kw OR 'ovarian cancer mutation':ti,ab,kw OR 'brca1- and brca2-associated hereditary breast and ovarian cancer':ti,ab,kw OR 'brca1- and brca2-associated hereditary breast-ovarian cancer':ti,ab,kw	19510

prophylaxis':ti,ab,kw OR 'prevention intervention':ti,ab,kw OR 'bilateral salpingo-oophorectomy':ti,ab,kw OR 'prophylactic bilateral mastectomy':ti,ab,kw OR 'bilateral mastectomy':ti,ab,kw OR 'preventive':ti,ab,kw OR 'preventive measures':ti,ab,kw OR 'surgery':ti,ab,kw OR 'risk-reducing mastectomy':ti,ab,kw OR 'risk-reducing strategy':ti,ab,kw OR 'risk-reducing strategies':ti,ab,kw OR 'risk-reducing medication':ti,ab,kw OR 'risk-reducing medications':ti,ab,kw OR 'bilateral risk-reducing mastectomy':ti,ab,kw OR 'estrogen receptor-positive':ti,ab,kw OR 'total mastectomy':ti,ab,kw OR 'nipple-sparing mastectomy':ti,ab,kw OR 'skin-sparing mastectomy':ti,ab,kw OR 'tamoxifen':ti,ab,kw OR 'raloxifene':ti,ab,kw OR 'aromatase inhibitors':ti,ab,kw OR 'anastrozole':ti,ab,kw OR 'exemestane':ti,ab,kw OR 'risk-reducing bilateral salpingo-oophorectomy':ti,ab,kw OR 'prophylactic bilateral salpingo-oophorectomy':ti,ab,kw OR 'screening':ti,ab,kw OR 'screen':ti,ab,kw OR 'testing':ti,ab,kw OR 'test':ti,ab,kw OR 'fact':ti,ab,kw OR 'chemoprevention':ti,ab,kw OR 'surveillance':ti,ab,kw OR 'breast cancer screening':ti,ab,kw OR 'prophylactic salpingectomy':ti,ab,kw OR 'selective oestrogen receptor modulators':ti,ab,kw OR 'arzoxifene':ti,ab,kw OR 'lasofoxifene':ti,ab,kw OR 'prophylactic oophorectomy':ti,ab,kw OR 'breast self-examination':ti,ab,kw OR 'annual clinical breast examination':ti,ab,kw OR 'semiannual clinical breast examination':ti,ab,kw OR 'annual mammography':ti,ab,kw OR 'breast mri':ti,ab,kw OR 'transvaginal ultrasound':ti,ab,kw OR 'serum ca-125':ti,ab,kw OR 'serum prostate-specific antigen':ti,ab,kw OR 'digital rectal exam screening':ti,ab,kw OR 'genetic counseling':ti,ab,kw OR 'genetic counseling':ti,ab,kw OR 'facilitated cascade testing':ti,ab,kw	6976681
#1 AND #2	10843
#3 AND 'human'/de	10236
#4 AND ('cancer model'/de OR 'case control study'/de OR 'clinical article'/de OR 'clinical audit'/de OR 'clinical study'/de OR 'clinical trial'/de OR 'cohort analysis'/de OR 'comparative effectiveness'/de OR 'comparative study'/de OR 'control group'/de OR 'controlled clinical trial'/de OR 'controlled clinical trial topic'/de OR 'controlled study'/de OR 'correlational study'/de OR 'cross sectional study'/de OR 'diagnostic test accuracy study'/de OR 'double blind procedure'/de OR 'drug surveillance program'/de OR 'evidence based medicine'/de OR 'evidence based practice'/de OR 'evidence based practice center'/de OR 'ex vivo study'/de OR 'experimental study'/de OR 'family study'/de OR 'genetic model'/de OR 'health belief model'/de OR 'human experiment'/de OR 'in vitro study'/de OR 'in vivo study'/de OR 'intervention study'/de OR 'linear regression analysis'/de OR 'logistic regression analysis'/de OR 'longitudinal study'/de OR 'multicenter study'/de OR 'multicenter study topic'/de OR 'multinomial logistic regression'/de OR 'normal human'/de OR 'observational study'/de OR 'outcomes research'/de OR 'panel study'/de OR 'participant observation'/de OR 'phase 1 clinical trial'/de OR 'phase 1 clinical trial topic'/de OR 'phase 2 clinical trial'/de OR 'phase 2 clinical trial topic'/de OR 'phase 3 clinical trial'/de OR 'phase 3 clinical trial topic'/de OR 'phase 4 clinical trial'/de OR 'pilot study'/de OR 'population based case control study'/de OR 'population model'/de OR 'postmarketing surveillance'/de OR 'preclinical study'/de OR 'prevention study'/de OR 'proportional hazards model'/de OR 'prospective study'/de OR 'quasi experimental study'/de OR 'randomized controlled trial'/de OR 'randomized	6397

	controlled trial topic'/de OR 'regression model'/de OR 'retrospective study'/de OR 'secondary analysis'/de OR 'single blind procedure'/de OR 'statistical model'/de OR 'theoretical model'/de OR 'theoretical study'/de OR 'trend study'/de OR 'tumor model'/de OR 'twin study'/de)	
Database name	Cochrane	Number
Date searched	Search conducted 13/10/23	1389
	(hereditary breast and ovarian cancer syndromes):ti,ab,kw OR (hereditary breast cancer syndrome):ti,ab,kw OR (hboc syndrome):ti,ab,kw OR (brca):ti,ab,kw OR (brca1 carrier):ti,ab,kw (Word variations have been searched)	1199
	(brca2 carrier):ti,ab,kw OR (hereditary breast-ovarian cancer syndrome):ti,ab,kw OR (hereditary breast-ovarian cancer):ti,ab,kw OR (hereditary breast and ovarian cancer syndrome):ti,ab,kw OR (hereditary breast and ovarian cancer):ti,ab,kw	187
	(genetic variation breast cancer):ti,ab,kw OR (breast cancer mutation):ti,ab,kw OR (brca mutation):ti,ab,kw OR (genetic variation ovarian cancer):ti,ab,kw OR (ovarian cancer mutation):ti,ab,kw	1882
	(prophylaxis):ti,ab,kw OR (prevention intervention):ti,ab,kw OR (bilateral salpingo-oophorectomy):ti,ab,kw OR (Prophylactic bilateral salpingo-oophorectomy):ti,ab,kw OR (prophylactic bilateral mastectomy):ti,ab,kw	88673
	(preventive):ti,ab,kw OR (preventive measures):ti,ab,kw OR (surgery):ti,ab,kw OR (risk-reducing mastectomy):ti,ab,kw OR (risk-reducing strategy):ti,ab,kw	285197
	(risk-reducing strategies):ti,ab,kw OR (risk-reducing medication):ti,ab,kw OR (risk-reducing medications):ti,ab,kw OR (bilateral risk-reducing mastectomy):ti,ab,kw OR (estrogen receptor-positive):ti,ab,kw	1853
	(total mastectomy):ti,ab,kw OR (nipple-sparing mastectomy):ti,ab,kw OR (skin-sparing mastectomy):ti,ab,kw OR (tamoxifen):ti,ab,kw OR (raloxifene):ti,ab,kw	7742
	(aromatase inhibitors):ti,ab,kw OR (anastrozole):ti,ab,kw OR (exemestane):ti,ab,kw OR (risk-reducing bilateral salpingo-oophorectomy):ti,ab,kw OR (prophylactic bilateral salpingo-oophorectomy):ti,ab,kw	3178
	(screening):ti,ab,kw OR (screen):ti,ab,kw OR (testing):ti,ab,kw OR (test):ti,ab,kw OR (fact):ti,ab,kw	413294
	(chemoprevention):ti,ab,kw OR (surveillance):ti,ab,kw OR (breast cancer screening):ti,ab,kw OR (prophylactic salpingectomy):ti,ab,kw OR (selective oestrogen receptor modulators):ti,ab,kw	15568
	(arzoxifene):ti,ab,kw OR (lasofoxifene):ti,ab,kw OR (prophylactic oophorectomy):ti,ab,kw OR (breast self-examination):ti,ab,kw OR (annual clinical breast examination):ti,ab,kw	504

	(semiannual clinical breast examination):ti,ab,kw OR (annual mammography):ti,ab,kw OR (breast mri):ti,ab,kw OR (transvaginal ultrasound):ti,ab,kw OR (serum ca-125):ti,ab,kw	2953
	(serum prostate-specific antigen):ti,ab,kw OR (digital rectal exam screening):ti,ab,kw OR (genetic counseling):ti,ab,kw OR (genetic counseling):ti,ab,kw OR (facilitated cascade testing):ti,ab,kw	2084
	#1 OR #2 OR #3	2377
	#4 OR #5 OR #6 OR #7 OR #8 OR #9 OR #10 OR #11 OR #12 OR #13	713170
	#14 AND #15 in Cochrane Protocols, Trials, Clinical Answers, Special Collections	1389

Database name	Pubmed	Number
Date searched	Search conducted 13/10/23	109
Search Strategy	((hereditary breast cancer[MeSH Terms]) OR (hereditary ovarian cancer [MeSH Terms])<br>OR (hereditary breast and ovarian cancer[MeSH Terms]) OR(BRCA) OR(BRCA1 carrier))<br>AND ((prophylaxis) OR (bilateral salpingo-oophorectomy) OR(Prophylactic bilateral salpingo-oophorectomy)<br>OR(prophylactic bilateral mastectomy) OR(bilateral mastectomy) OR(risk-reducing mastectomy)<br>OR (Bilateral risk-reducing mastectomy) OR (elective bilateral risk-reducing mastectomy)<br>OR (elective estrogen receptor modulators) OR (estrogen receptor-positive) OR(total mastectomy)<br>OR (nipple-sparing mastectomy) OR(skin-sparing mastectomy) OR(tamoxifen) OR(raloxifene)<br>OR(aromatase inhibitors) OR(anastrozole) OR(exemestane) OR(risk-reducing bilateral salpingo-oophorectomy)<br>OR (Prophylactic bilateral salpingo-oophorectomy) OR(screening) OR(screen) OR(testing) OR(test) OR(FaCT))<br>AND ((randomized controlled trial [Title]) OR(cohort study[Title]) OR (retrospective study[Title]))	109

Database name	Ovid	Number
Date searched	Search conducted 13/10/23	896
Search Strategy	1. Hereditary Breast Cancer.mp.<br>2. Hereditary Ovarian Cancer.mp.<br>3. Hereditary Breast Cancer Syndrome.mp.<br>4. HBOC syndrome.mp.<br>5. BRCA.mp.	

6. BRCA1 carrier.mp.
7. BRCA2 carrier.mp.
8. Hereditary Breast-ovarian Cancer Syndrome.mp.
9. Hereditary Breast-ovarian Cancer.mp.
10. (Hereditary Breast and ovarian Cancer Syndrome).mp.
11. (Hereditary Breast and ovarian Cancer).mp.
12. genetic variation breast cancer.mp.
13. breast cancer mutation.mp.
14. BRCA mutation.mp.
15. genetic variation ovarian cancer.mp.
16. ovarian cancer mutation.mp.
17. (BRCA1- and BRCA2-Associated Hereditary Breast and Ovarian Cancer).mp.
18. (BRCA1- and BRCA2-Associated Hereditary Breast-Ovarian Cancer).mp.
19. prophylaxis.mp.
20. prevention intervention.mp.
21. bilateral salpingo-oophorectomy.mp.
22. Prophylactic bilateral salpingo-oophorectomy.mp.
23. prophylactic bilateral mastectomy.mp.
24. bilateral mastectomy.mp.
25. preventive.mp.
26. preventive measures.mp.
27. surgery.mp.
28. risk-reducing mastectomy.mp.
29. risk-reducing strategy.mp.
30. risk-reducing strategies.mp.
31. risk-reducing medication.mp.
32. risk-reducing medications.mp.
33. Bilateral risk-reducing mastectomy.mp.
34. estrogen receptor-positive.mp.
35. total mastectomy.mp.
36. nipple-sparing mastectomy.mp.
37. skin-sparing mastectomy.mp.
38. tamoxifen.mp.
39. raloxifene.mp.
40. aromatase inhibitors.mp.
41. anastrozole.mp.
42. exemestane.mp.
43. risk-reducing bilateral salpingo-oophorectomy.mp.
44. Prophylactic bilateral salpingo-oophorectomy.mp.
45. screening.mp.
46. screen.mp.
47. testing.mp.
48. test.mp.
49. FaCT.mp.
50. chemoprevention.mp.
51. surveillance.mp.
52. Breast cancer screening.mp.
53. Prophylactic salpingectomy.mp.
54. Selective oestrogen receptor modulators.mp.
55. arzoxifene.mp.
56. lasofoxifene.mp.
57. Prophylactic oophorectomy.mp.

58. breast self-examination.mp.
59. annual clinical breast examination.mp.
60. semiannual clinical breast examination.mp.
61. annual mammography.mp.
62. breast MRI.mp.
63. transvaginal ultrasound.mp.
64. serum CA-125.mp.
65. serum prostate-specific antigen.mp.
66. digital rectal exam screening.mp.
67. Genetic Counseling.mp.
68. cascade testing.mp.
69. Facilitated cascade testing.mp.
70. cancer model.ti,ab.
71. case control study.ti,ab.
72. clinical article.ti,ab.
73. clinical audit.ti,ab.
74. clinical study.ti,ab.
75. clinical trial.ti,ab.
76. cohort analysis.ti,ab.
77. comparative effectiveness.ti,ab.
78. comparative study.ti,ab.
79. control group.ti,ab.
80. controlled clinical trial.ti,ab.
81. ontrolled clinical trial topic.ti,ab.
82. controlled study.ti,ab.
83. correlational study.ti,ab.
84. controlled clinical trial topic.ti,ab.
85. cross sectional study.ti,ab.
86. diagnostic test accuracy study.ti,ab.
87. double blind procedure.ti,ab.
88. drug surveillance program.ti,ab.
89. evidence based medicine.ti,ab.
90. evidence based practice.ti,ab.
91. evidence based practice center.ti,ab.
92. family study.ti,ab.
93. genetic model.ti,ab.
94. health belief model.ti,ab.
95. human experiment.ti,ab.
96. in vitro study.ti,ab.
97. in vivo study.ti,ab.
98. intervention study.ti,ab.
99. linear regression analysis.ti,ab.
100. logistic regression analysis.ti,ab.
101. longitudinal study.ti,ab.
102. multicenter study.ti,ab.
103. multicenter study topic.ti,ab.
104. multinomial logistic regression.ti,ab.
105. normal human.ti,ab.
106. observational study.ti,ab.
107. outcomes research.ti,ab.
108. panel study.ti,ab.
109. participant observation.ti,ab.

110. phase 1 clinical trial.ti,ab.
111. phase 1 clinical trial topic.ti,ab.
112. phase 2 clinical trial.ti,ab.
113. phase 2 clinical trial topic.ti,ab.
114. phase 3 clinical trial.ti,ab.
115. phase 3 clinical trial topic.ti,ab.
116. phase 4 clinical trial.ti,ab.
117. pilot study.ti,ab.
118. population based case control study.ti,ab.
119. population model.ti,ab.
120. postmarketing surveillance.ti,ab.
121. preclinical study.ti,ab.
122. prevention study.ti,ab.
123. proportional hazards model.ti,ab.
124. prospective study.ti,ab.
125. quasi experimental study.ti,ab.
126. randomized controlled trial.ti,ab.
127. randomized controlled trial topic.ti,ab.
128. regression model.ti,ab.
129. retrospective study.ti,ab.
130. secondary analysis.ti,ab.
131. single blind procedure.ti,ab.
132. statistical model.ti,ab.
133. theoretical model.ti,ab.
134. theoretical study.ti,ab.
135. trend study.ti,ab.
136. tumor model.ti,ab.
137. twin study.ti,ab.
138. 1 or 2 or 3 or 4 or 5 or 6 or 7 or 8 or 9 or 10 or 11 or 12 or 13 or 14 or 15 or 16 or 17 or 18
139. 19 or 20 or 21 or 22 or 23 or 24 or 25 or 26 or 27 or 28 or 29 or 30 or 31 or 32 or 33 or 34 or 35 or 36 or 37 or 38 or 39 or 40 or 41 or 42 or 43 or 44 or 45 or 46 or 47 or 48 or 49 or 50 or 51 or 52 or 53 or 54 or 55 or 56 or 57 or 58 or 59 or 60 or 61 or 62 or 63 or 64 or 65 or 66 or 67 or 68 or 69
140. 70 or 71 or 72 or 73 or 74 or 75 or 76 or 77 or 78 or 79 or 80 or 81 or 82 or 83 or 84 or 85 or 86 or 87 or 88 or 89 or 90 or 91 or 92 or 93 or 94 or 95 or 96 or 97 or 98 or 99 or 100 or 101 or 102 or 103 or 104 or 105 or 106 or 107 or 108 or 109 or 110 or 111 or 112 or 113 or 114 or 115 or 116 or 117 or 118 or 119 or 120 or 121 or 122 or 123 or 124 or 125 or 126 or 127 or 128 or 129 or 130 or 131 or 132 or 133 or 134 or 135 or 136 or 137
141. 138 and 139 and 140
142. limit 141 to english language

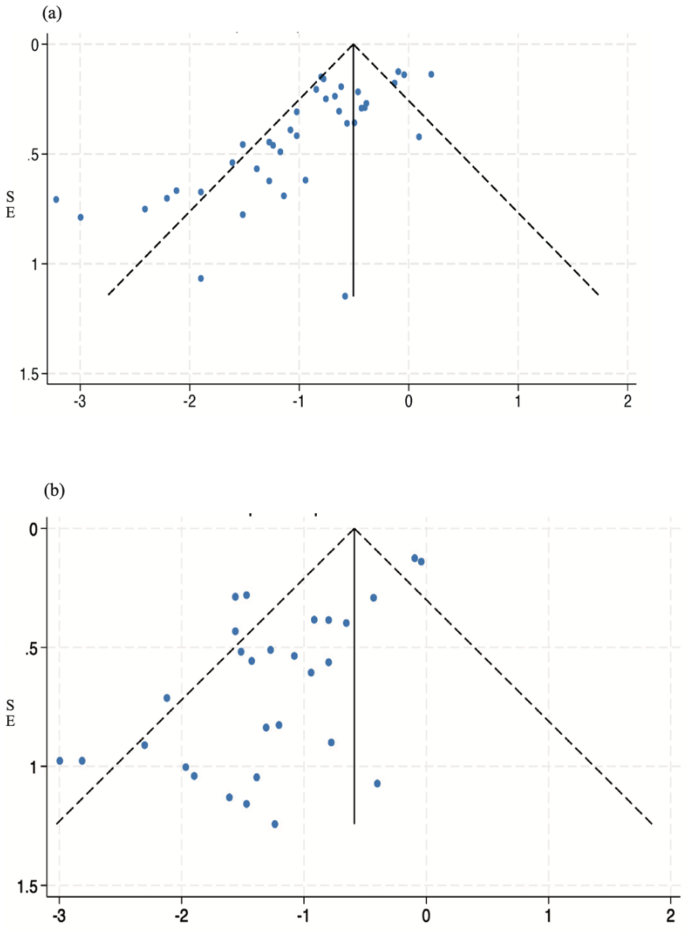

Figure S1. Funnel Plot.

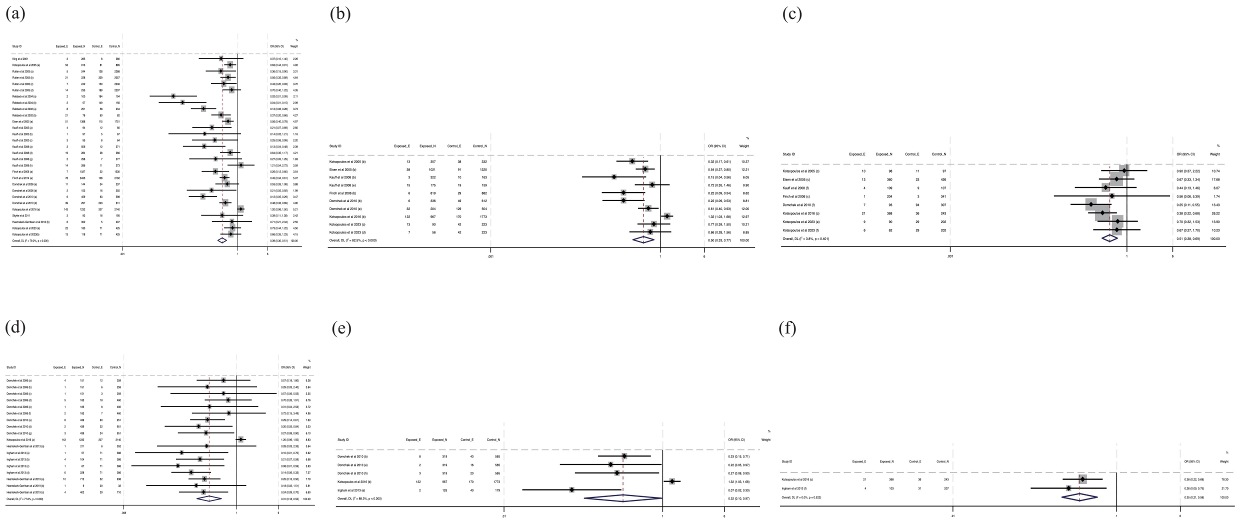

Figure S2. Self-calculate forest plots of subgroup analysis for intervention for BRCA1/2, BRCA1, and BRCA2 mutation carriers [1-18].

Notes: Panel (a) provides a synthesis of odds ratios for cancer risk reduction in all BRCA mutations. Panel (b) displays odds ratios for disease risk associated with BRCA1 mutations. Panel (c) presents odds ratios for disease risk linked to BRCA2 mutations. Panels (d), (e), and (f) illustrate the synthesis of ORs for mortality risk reduction corresponding to all BRCA mutations, BRCA1 mutations, and BRCA2 mutations, respectively.

(a)

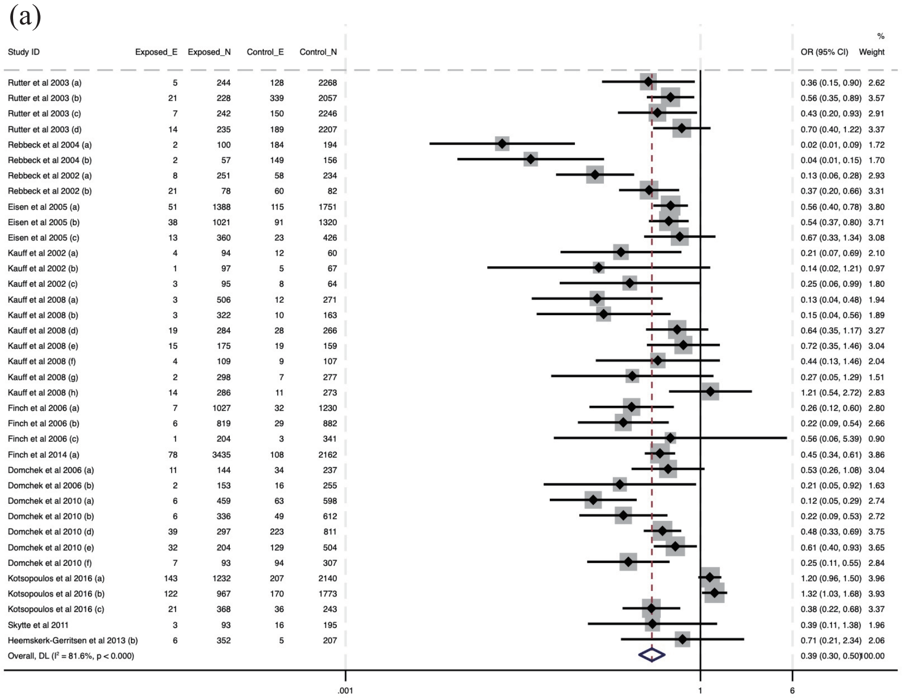

(b)

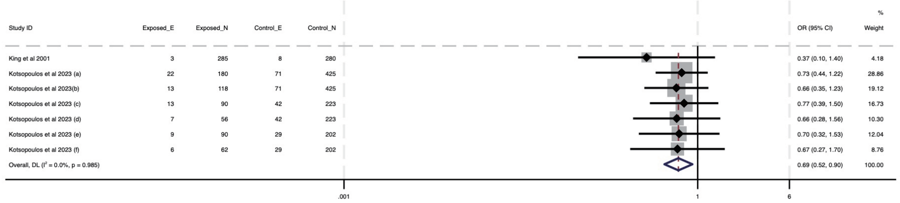

Figure S3. Self-calculate forest plots of subgroup analysis for effectiveness of RRS and chemoprevention [3-16]

Notes: Panel (a) presents the effectiveness of RRS for cancer risk and panel (b) presents the effectiveness of chemoprevention for cancer risk.

(a)

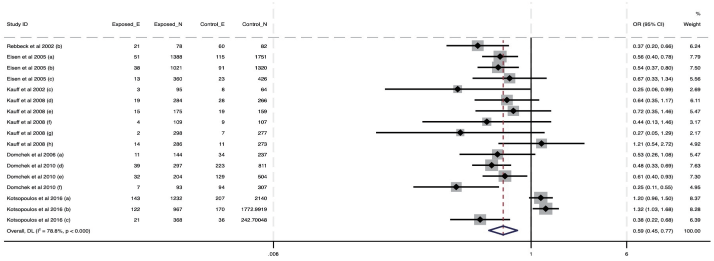

(b)

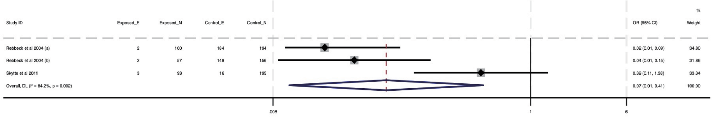

(c)

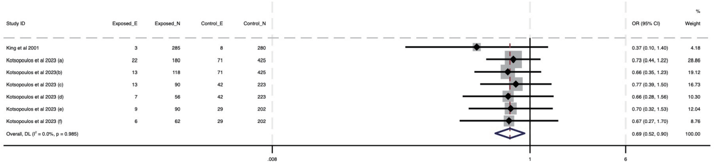

(d)

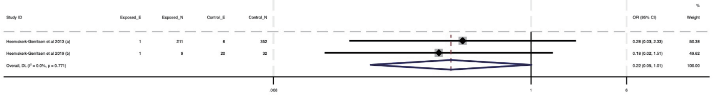

(e)

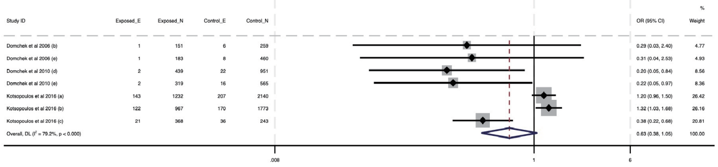

Figure S4. Self-calculate forest plots of different interventions effectiveness in BC-specific cancer risk and mortality [4-8, 11-16]

Notes: Panel (a) indicates the effectiveness of oophorectomy in BC-specific cancer risk reduction. Panel (b) indicates the effectiveness of mastectomy in BC-specific cancer risk reduction. Panel (c) indicates the effectiveness of chemoprevention in BC-specific cancer risk reduction. Panel (d) indicates the effectiveness of mastectomy in BC-specific mortality risk reduction. Panel (e) indicates the effectiveness of oophorectomy in BC-specific mortality risk reduction.

(a)

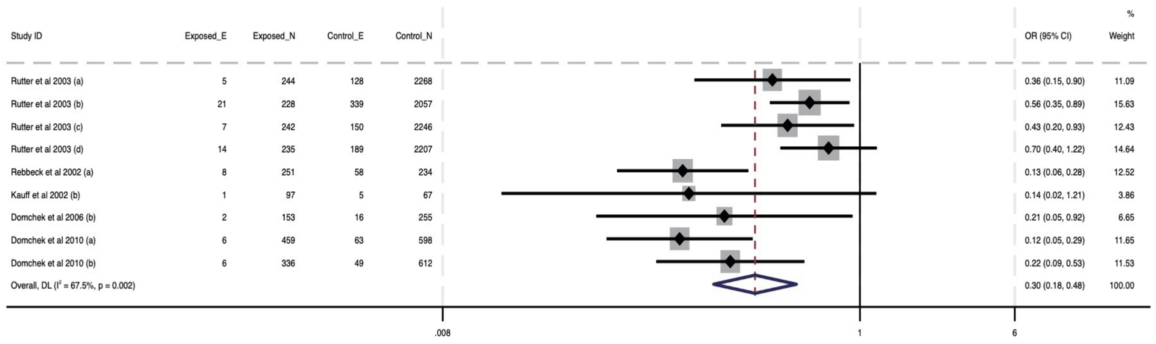

(b)

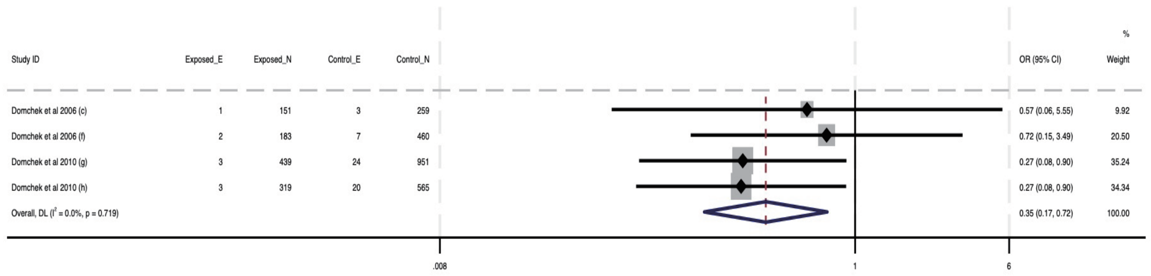

Figure S5. Self-calculated forest plots of effectiveness of interventions in OC-specific cancer risk and mortality risk reduction [3, 5, 7, 11, 12]

Notes: Panel (a) indicates the effectiveness of oophorectomy in OC-specific cancer risk reduction and panel (b) indicates the effectiveness of oophorectomy in OC-specific mortality risk reduction.

Reference

1. King, M.-C.; Wieand, S.; Hale, K.; Lee, M.; Walsh, T.; Owens, K.; Tait, J.; Ford, L.; Dunn, B.K.; Costantino, J. Tamoxifen and breast cancer incidence among women with inherited mutations in BRCA1 and BRCA2: National Surgical Adjuvant Breast and Bowel Project (NSABP-P1) Breast Cancer Prevention Trial. *JAMA* **2001**, *286*, 2251–2256.
2. Kotsopoulos, J.; Olopade, O.I.; Ghadirian, P.; Lubinski, J.; Lynch, H.T.; Isaacs, C.; Weber, B.; Kim-Sing, C.; Ainsworth, P.; Foulkes, W.D. Changes in body weight and the risk of breast cancer in BRCA1 and BRCA2 mutation carriers. *Breast Cancer Res.* **2005**, *7*, 1–11.
3. Rutter, J.L.; Wacholder, S.; Chetrit, A.; Lubin, F.; Menczer, J.; Ebbers, S.; Tucker, M.A.; Struewing, J.P.; Hartge, P. Gynecologic surgeries and risk of ovarian cancer in women with BRCA1 and BRCA2 Ashkenazi founder mutations: An Israeli population-based case–control study. *J. Natl. Cancer Inst.* **2003**, *95*, 1072–1078.
4. Rebbeck, T.R.; Friebel, T.; Lynch, H.T.; Neuhausen, S.L.; Van't Veer, L.; Garber, J.E.; Evans, G.R.; Narod, S.A.; Isaacs, C.; Matloff, E. Bilateral prophylactic mastectomy reduces breast cancer risk in BRCA1 and BRCA2 mutation carriers: The PROSE Study Group. *J. Clin. Oncol.* **2004**, *22*, 1055–1062.
5. Rebbeck, T.R.; Lynch, H.T.; Neuhausen, S.L.; Narod, S.A.; Van't Veer, L.; Garber, J.E.; Evans, G.; Isaacs, C.; Daly, M.B.; Matloff, E. Prophylactic oophorectomy in carriers of BRCA1 or BRCA2 mutations. *N. Engl. J. Med.* **2002**, *346*, 1616–1622.
6. Eisen, A.; Lubinski, J.; Klijn, J.; Moller, P.; Lynch, H.T.; Offit, K.; Weber, B.; Rebbeck, T.; Neuhausen, S.L.; Ghadirian, P. Breast cancer risk following bilateral oophorectomy in BRCA1 and BRCA2 mutation carriers: An international case-control study. *J. Clin. Oncol.* **2005**, *23*, 7491–7496.
7. Kauff, N.D.; Satagopan, J.M.; Robson, M.E.; Scheuer, L.; Hensley, M.; Hudis, C.A.; Ellis, N.A.; Boyd, J.; Borgen, P.I.; Barakat, R.R. Risk-reducing salpingo-oophorectomy in women with a BRCA1 or BRCA2 mutation. *N. Engl. J. Med.* **2002**, *346*, 1609–1615.
8. Kauff, N.D.; Domchek, S.M.; Friebel, T.M.; Robson, M.E.; Lee, J.; Garber, J.E.; Isaacs, C.; Evans, D.G.; Lynch, H.; Eeles, R.A. Risk-reducing salpingo-oophorectomy for the prevention of BRCA1-and BRCA2-associated breast and gynecologic cancer: A multicenter, prospective study. *J. Clin. Oncol.* **2008**, *26*, 1331.
9. Finch, A.; Beiner, M.; Lubinski, J.; Lynch, H.T.; Moller, P.; Rosen, B.; Murphy, J.; Ghadirian, P.; Friedman, E.; Foulkes, W.D. Salpingo-oophorectomy and the risk of ovarian, fallopian tube, and peritoneal cancers in women with a BRCA1 or BRCA2 Mutation. *JAMA* **2006**, *296*, 185–192.
10. Finch, A.P.; Lubinski, J.; Møller, P.; Singer, C.F.; Karlan, B.; Senter, L.; Rosen, B.; Maehle, L.; Ghadirian, P.; Cybulski, C. Impact of oophorectomy on cancer incidence and mortality in women with a BRCA1 or BRCA2 mutation. *J. Clin. Oncol.* **2014**, *32*, 1547.
11. Domchek, S.M.; Friebel, T.M.; Neuhausen, S.L.; Wagner, T.; Evans, G.; Isaacs, C.; Garber, J.E.; Daly, M.B.; Eeles, R.; Matloff, E. Mortality after bilateral salpingo-oophorectomy in BRCA1 and BRCA2 mutation carriers: A prospective cohort study. *Lancet Oncol.* **2006**, *7*, 223–229.
12. Domchek, S.M.; Friebel, T.M.; Singer, C.F.; Evans, D.G.; Lynch, H.T.; Isaacs, C.; Garber, J.E.; Neuhausen, S.L.; Matloff, E.; Eeles, R. Association of risk-reducing surgery in BRCA1 or BRCA2 mutation carriers with cancer risk and mortality. *JAMA* **2010**, *304*, 967–975.
13. Kotsopoulos, J.; Huzarski, T.; Gronwald, J.; Singer, C.F.; Moller, P.; Lynch, H.T.; Armel, S.; Karlan, B.; Foulkes, W.D.; Neuhausen, S.L. Bilateral oophorectomy and breast cancer risk in BRCA1 and BRCA2 mutation carriers. *J. Natl. Cancer Inst.* **2017**, *109*, djw177.
14. Skytte, A.B.; Crüger, D.; Gerster, M.; Lænkholm, A.V.; Lang, C.; Brøndum-Nielsen, K.; Andersen, M.; Sunde, L.; Kølvraa, S.; Gerdes, A.M. Breast cancer after bilateral risk-reducing mastectomy. *Clin. Genet.* **2011**, *79*, 431–437.
15. Heemskerk-Gerritsen, B.; Menke-Pluijmers, M.; Jager, A.; Tilanus-Linthorst, M.; Koppert, L.; Obdeijn, I.; Van Deurzen, C.; Collée, J.; Seynaeve, C.; Hooning, M. Substantial breast cancer risk reduction and potential survival benefit after bilateral mastectomy when compared with surveillance in healthy BRCA1 and BRCA2 mutation carriers: A prospective analysis. *Ann. Oncol.* **2013**, *24*, 2029–2035.
16. Kotsopoulos, J.; Gronwald, J.; Huzarski, T.; Aeilts, A.; Randall Armel, S.; Karlan, B.; Singer, C.F.; Eisen, A.; Tung, N.; Olopade, O. Tamoxifen and the risk of breast cancer in women with a BRCA1 or BRCA2 mutation. *Breast Cancer Res. Treat.* **2023**, *201*, 257–264.
17. Ingham, S.L.; Sperrin, M.; Baildam, A.; Ross, G.L.; Clayton, R.; Lalloo, F.; Buchan, I.; Howell, A.; Evans, D.G.R. Risk-reducing surgery increases survival in BRCA1/2 mutation carriers unaffected at time of family referral. *Breast Cancer Res. Treat.* **2013**, *142*, 611–618.
18. Heemskerk-Gerritsen, B.A.; Jager, A.; Koppert, L.B.; Obdeijn, A.I.-M.; Collée, M.; Meijers-Heijboer, H.E.; Jenner, D.J.; Oldenburg, H.S.; van Engelen, K.; de Vries, J. Survival after bilateral risk-reducing mastectomy in healthy BRCA1 and BRCA2 mutation carriers. *Breast Cancer Res. Treat.* **2019**, *177*, 723–733.
